# Psychological interventions to improve glycemic control in adults with type 2 diabetes: a systematic review and meta-analysis

**DOI:** 10.1136/bmjdrc-2019-001150

**Published:** 2020-04-08

**Authors:** Kirsty Winkley, Rebecca Upsher, Daniel Stahl, Daniel Pollard, Alan Brennan, Simon R Heller, Khalida Ismail

**Affiliations:** 1 Florence Nightingale Faculty of Nursing, Midwifery & Palliative Care, King’s College London, London, UK; 2 Institute of Psychiatry, Psychology & Neuroscience, King’s College London, London, UK; 3 Department of Biostatistics, King’s College London, London, UK; 4 School of Health and Related Research, Health Economics and Decision Science, University of Sheffield, Sheffield, UK; 5 Department of Oncology & Metabolism, University of Sheffield, Sheffield, UK

**Keywords:** type 2 diabetes, psychology, randomized controlled trial, meta-analysis

## Abstract

The quality of evidence that psychological interventions are effective in improving glycemic control in adults with type 2 diabetes (T2D) is weak.

We conducted a systematic review and meta-analysis of psychological interventions in T2D to assess whether their effectiveness in improving glycemic levels has improved over the past 30 years. We applied the protocol of a systematic review and aggregate meta-analysis conducted to January 2003. We added network meta-analysis (NMA) to compare intervention and control group type against usual care. MEDLINE, Cumulative Index to Nursing and Allied Health Literature, PsycINFO, EMBASE, Cochrane Controlled Trials Database, Web of Science, and Dissertation Abstracts International were searched from January 2003 to July 2018. Only randomized controlled trials (RCT) of psychological interventions for adults with T2D reported in any language were included. The primary outcome was change in glycemic control (glycated hemoglobin (HbA1c) in mmol/mol). Data were extracted from study reports and authors were contacted for missing data.

94 RCTs were eligible for inclusion in the systematic review since the last review. In 70 RCTs (n=14 796 participants) the pooled mean difference in HbA1c in those randomized to psychological intervention compared with control group was −0.19 (95% CI −0.25 to −0.12), equivalent to a reduction in HbA1c of 3.7 mmol/mol, with moderate heterogeneity across studies (I^2^=64.7%, p<0.001). NMA suggested the probability of intervention effectiveness is highest for self-help materials, cognitive–behavioral therapy, and counseling, compared with usual care. Limitations of this study include that there is a possibility that some studies may have been missed if diabetes did not appear in the title or abstract.

The effectiveness of psychological interventions for adults with T2D have minimal clinical benefit in improving glycemic control.

**PROSPERO registration number:**

CRD42016033619.

## Introduction

In type 2 diabetes (T2D), management involves adopting multiple self-care tasks which include consuming lower energy dense diets, increasing physical activity, self-administration of oral and injectable therapies, self-monitoring of blood glucose levels, decision-making about dose of insulin, and attending education and annual review appointments.

Despite evidence-based guidelines,^1^ at least a third of people with T2D do not achieve target glycated hemoglobin (HbA1c) levels.^2^ This is partly attributable to psychological factors that adversely affect self-management. These include depressive disorders,^3^ anxiety disorders,^4^ and diabetes-specific distress, such as fear of diabetes complications, hypoglycemia,^5^ insulin,^6^ disordered eating,^7^ the burden of living with T2D and stigma.^8^ Psychological treatments, such as cognitive–behavioral therapy (CBT) and counseling, including motivational interviewing, are offered with the aim of improving self-management. In 2003, we conducted a systematic review and meta-analysis of randomized controlled trials (RCTs) testing the effectiveness of psychological interventions in improving glycemic control in T2D. We found that there was a small but clinically significant reduction in HbA1c by 8 mmol/mol.^9^ At that time, there were only 12 studies with a pooled sample of 522 participants, and most were published before the Consolidated Standards of Reporting Trials (CONSORT)^10^ and were of low methodological quality. Since then guidance for conducting and reporting complex interventions has been published and widely disseminated, and there has been an explosion in the number of RCTs. The aim was to conduct a systematic review and meta-analysis to assess the effectiveness of psychological treatments as compared with control conditions in improving glycemic control in adults with T2D and whether the strength for the association was improving over time.

## Methods

We repeated the original protocol for the systematic review and aggregate meta-analysis for the primary outcome, the change in HbA1c.^9^ We added network meta-analysis (NMA) to enable us to compare all intervention arms and attention control groups with usual care and expanded the data extraction to include further details about the intervention that allow for potential replication; the protocol is available at https://www.journalslibrary.nihr.ac.uk/programmes/hta/1421310#/.^11^ ^12^ The Preferred Reporting Items for Systematic Reviews and Meta-Analyses (PRISMA) statement^13^ and relevant extensions were followed.

### Data sources and searches

MEDLINE (OVID), Cumulative Index to Nursing and Allied Health Literature, PsycINFO, EMBASE (OVID), Cochrane Controlled Trials Database, Web of Science, and Dissertation Abstracts International were searched from 1 January 2003 to 1 July 2018. (Our earlier review searched literature from inception of electronic databases to January 2003.) Conference proceedings from Diabetes UK, American Diabetes Association, European Association for the Study of Diabetes, and International Diabetes Federation were searched for the past 5 years (from 2012 to 2018). We checked the US government trial registry (ClinicalTrials.gov) and searched publication status for any ongoing RCTs. Finally, the reference lists of the included studies and other reviews were searched for additional studies, and leading experts and investigators of ongoing RCTs identified from clinical trials registers were contacted for additional information. Web of Science (formerly Web of Knowledge), launched in 2002, and ClinicalTrials.gov became widely mandated from 2004 onwards, and Dissertation Abstracts International is a leading international repository since 2008; therefore, these data sources were additional sources to those specified in the original protocol.^9^

We used the Cochrane Collaboration’s optimum search strategy. The following terms were applied to search MEDLINE: ‘diabetes mellitus’, ‘psychological therapies’ and ‘mood disorders’, and ‘clinical trials’; these were adjusted for other databases (online supplementary appendix 1, online supplementary table S1). We included additional keywords for some newer therapies, such as ‘Acceptance Commitment Therapy (ACT)’ and ‘Mindfulness’.

10.1136/bmjdrc-2019-001150.supp1 Supplementary data

10.1136/bmjdrc-2019-001150.supp2 Supplementary data

### Study selection

Studies eligible for inclusion were RCTs of a psychological intervention as defined previously for adults (age 18 years and older) with T2D. There was no language restriction. Psychological interventions were categorized as supportive or counseling therapy, including motivational interviewing; CBT, including techniques commonly used in CBT, such as relaxation, cognitive restructuring, goal-setting, and problem-solving; and psychodynamic or interpersonal psychotherapy. We were mindful that newer therapies may have been developed and may not fall into these criteria. Studies which did not explicitly describe the intervention or techniques or which did not have face validity for these categories underwent consensus discussion by an academic liaison psychiatrist, health psychologist and nurse therapist trained in motivational interviewing (KI, RU, and KW, respectively). If agreement could not be reached, the study was excluded. Comparators were defined as usual care, waiting list, attention control (matching the number of sessions as in the intervention arm) and diabetes education.

The main outcome was change in glycemic control using HbA1c (mmol/mol) between baseline and follow-up (closest to 12 months). HbA1c was an inclusion criterion for the review.

All titles and abstracts of identified articles from the search were screened by two independent reviewers (RU and KW) to determine if they met the inclusion criteria. Full-text articles were accessed, and inter-rater reliability was conducted to determine agreement for inclusion. If there was a disagreement at title and abstract screening, the study was included for full-text screening. Quasi-RCT, N-of-1 and any design other than RCT were excluded.

### Data extraction and quality assessment

Data were extracted independently by both reviewers (RU and KW). The data extraction form was managed in Microsoft Excel, piloted independently on five included studies and compared among reviewers before applying to the rest of the studies. Studies written in a language other than English were translated and data were extracted by a native speaker. If there were multiple publications, the main one reporting the baseline and follow-up closest to 12 months was included. When studies involved more than one psychological treatment, data from the most intensive psychological treatment were included for the aggregate meta-analysis. Intensity was defined as the number and duration of sessions (hours) and the duration of therapy (months). Data from all allocations (including alternative intervention, eg, self-help materials and control treatments) were extracted for NMA. Missing data were requested from the authors. Any disagreements were discussed with a third reviewer (KI) until consensus was reached. We extracted data in a standardized format for country of origin and year. Data extracted on participant characteristics were summary estimates and included age, gender, ethnicity, glycemic control at baseline and at follow-up, duration of T2D, type of diabetes treatment, and duration of follow-up. When studies included type 1 and type 2 diabetes, only data on T2D were extracted if the data had been stratified by type. The characteristics of all interventions were coded as type, duration, number of sessions, mode of delivery (individual, group, family), therapist characteristics (profession), manualized treatment, and duration of follow-up. In line with developments in methodology for complex interventions,^11^ we extracted information on underpinning psychological theory and data describing fidelity to the intervention and competency of the therapist.^12^

We changed the quality assessment from the original protocol to the Cochrane Risk of Bias tool as this had greater validity^14^ in determining high, low or unclear risk of bias (RoB),^14^ within and across studies. RoB was conducted independently (RU and KW) and disagreements were resolved by a third reviewer (KI). A subgroup meta-analysis was conducted by RoB rating, and meta-regression compared effect sizes between RoB groups.

### Data synthesis and analysis

For the aggregate meta-analysis, the standardized mean difference (SMD), Cohen’s d, was calculated to determine change in HbA1c (mmol/mol) between baseline and 12-month follow-up or closest to that data point. SMDs were pooled in random-effects meta-analysis. SMDs were converted to absolute HbA1c values by multiplying SMD by pooled SD of all studies included in the meta-analysis. Diagnostic analyses included investigations of the effect of removing individual studies; Egger’s publication bias; and funnel plots^15^ and the trim and fill procedure^16^ to determine potential for missing studies. Meta-regression was conducted if there were five or more studies with data that could be pooled.^17^ All meta-analyses were conducted using STATA V.14.

Non-protocol analyses were performed. For example meta-regressions were performed for the association between HbA1c and the primary outcome category; HbA1c primary outcome (vs HbA1c secondary outcome); comorbid depression inclusion criteria (vs no comorbid depression criteria); and suboptimal HbA1c inclusion criteria (vs no suboptimal HbA1c inclusion criteria). In addition, meta-regressions were performed to determine the interaction between depressive symptoms as an inclusion criterion and whether HbA1c was the study’s primary or secondary outcome, and the interaction between studies with suboptimal HbA1c as an inclusion criterion and whether HbA1c was the study’s primary outcome.

To determine the potential for cohort effects, we linked the data from the original meta-analysis removing any duplicate studies.

For the NMA we analyzed direct and indirect effects of the treatment and control arms on the mean change in HbA1c.^18^ Indirect effects compared categories of intervention (psychological interventions, alternative treatments) or control groups (usual care, attention control, waiting list, diabetes education) within and across studies. We constructed network plots for direct comparisons. We conducted random-effects meta-analysis allowing for heterogeneity and inconsistency between the studies.^19 20^ Inconsistency was assessed by comparing direct and indirect effects of the contrast I-J and Wald tests. Hedges’ g formula was used to determine unbiased SMDs corrected for df for different categories of intervention with usual care as the control.^21^ Finally, we estimated potential ranks for each category using cumulative probability plots and surface under the cumulative ranking (SUCRA); the higher the SUCRA (closest to 1), the greater the probability of the intervention being effective.

## Results

### Study selection

**Figure 1:**
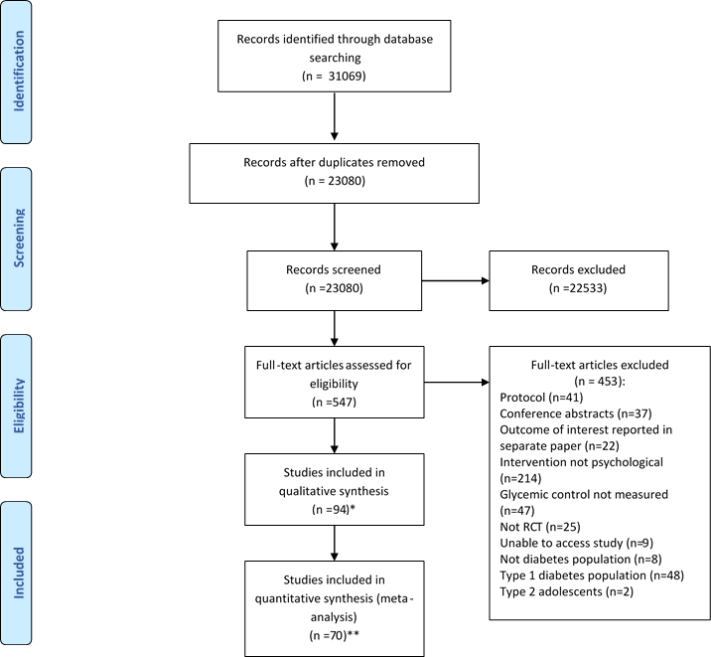
Qualitative and quantitative PRISMA flow chart for all type 2 diabetes studies. *Sixteen studies were papers which included a type 1 and type 2 diabetes population where separate analysis per diabetes type could not be obtained. In the remaining eight studies which were not included in the meta-analysis, not enough information for meta-analysis was reported in the paper and could not be provided by author when contacted. **Three studies had a type 1 and type 2 diabetes population where separate analysis per diabetes type was obtained. PRISMA, Preferred Reporting Items for Systematic Reviews and Meta-Analyses; RCT, randomized controlled trial.

We identified 31 069 study citations from the literature search (figure 1). Once duplicates were removed, titles and abstracts of 23 080 citations were screened, from which 547 full texts were selected for further extraction. There was 94.5% agreement in identifying abstracts for full retrieval (Cohen’s kappa=0.95). We identified 94 RCTs that met the inclusion criteria for the systematic review, and the reasons for exclusion of the other studies are shown in figure 1.

### Study characteristics

The studies included in the systematic review are listed and the study and intervention characteristics are synthesized in online supplementary table S2. There was a broad range of clinical settings and/or criteria, such as suboptimal glycemic control (n=28), specific duration of diabetes (n=19), age (n=41), body mass index (n=10) and depression (n=11). There were no RCTs administering psychodynamic therapy, while 33 RCTs administered CBT or techniques that fall under its umbrella such as relaxation therapy or problem-solving, 60 RCTs delivered counseling, and 1 used interpersonal psychotherapy (IPT). In the control group, there were 60, 20, 10 and 4 studies administering usual care, attention control, waiting list, and diabetes education, respectively. Most therapists were diabetes specialists (n=37) or psychologists (n=31), and others (n=26) defined as research assistants (n=10), non-diabetes health professionals (n=11), lay people (n=3), or did not report their profession (n=2). Most interventions were delivered face to face (n=75), and mostly to individuals (n=54) and groups (n=37). The mean number of therapy sessions offered was 7.41 (SD 4.60), the mean duration of each session was 1.40 hours (SD 1.03), and the mean duration of therapy was 5.44 months (SD 6.54). Twenty-seven studies referred to an intervention manual, of which 7 provided a link to the manual and 24 studies provided a link to the study protocol.

### RoB within studies

Of the studies included in the meta-analysis, few were assessed as high RoB (n=3). The majority of studies were either of low RoB (n=29) or unclear RoB (n=38) (online supplementary figure S1), and there was no association between these RoB categories and HbA1c (p=0.23).

### Results of individual studies

**Figure 2:**
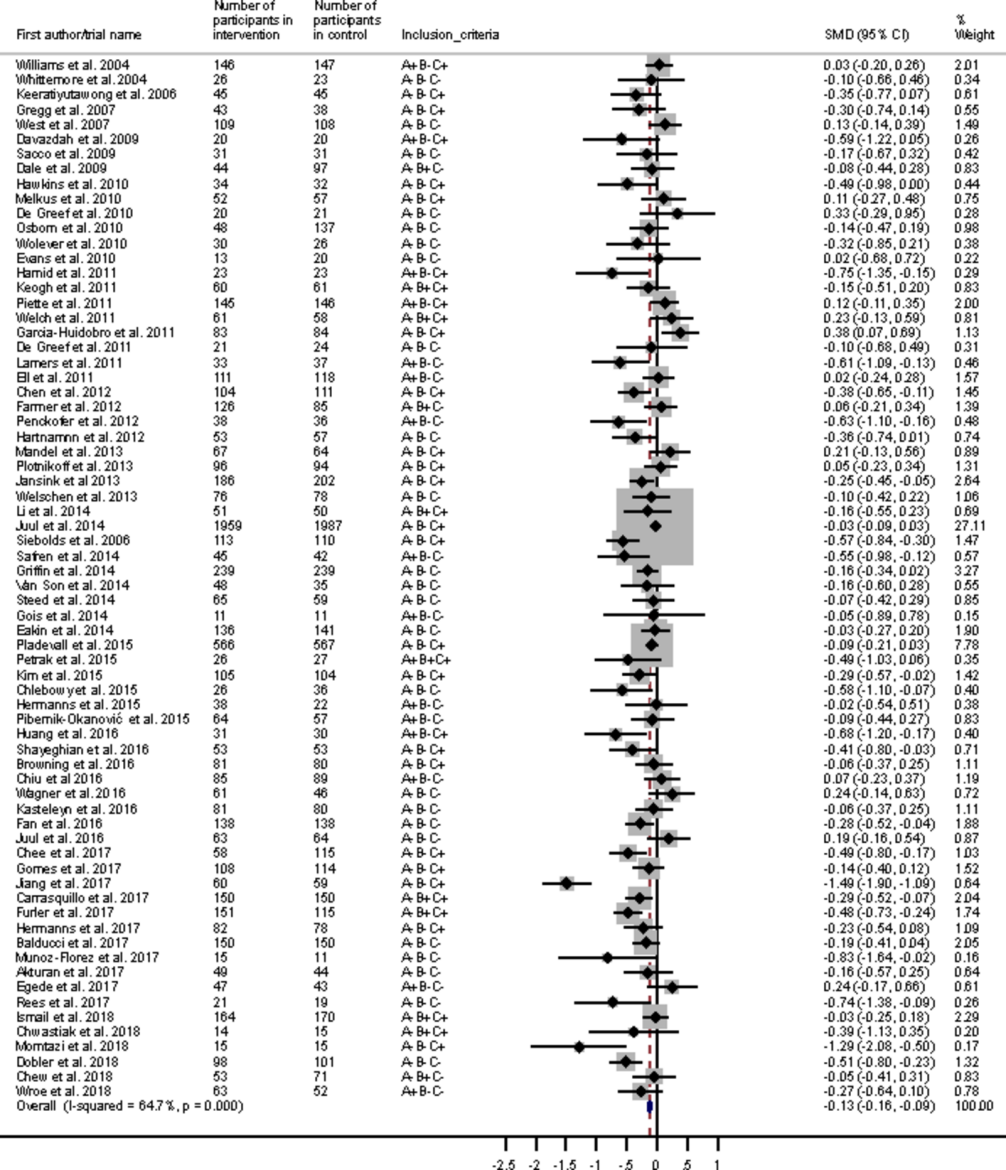
Forest plot for a random-effect meta-analysis of standardized mean difference in HbA1c comparing psychological intervention with control group for adults with type 2 diabetes. A+, depressive symptoms in inclusion criteria; A-, depressive symptoms not inclusion criteria; B+, suboptimal HbA1c in inclusion criteria (7.5%/58mmol/mol or more); B-, suboptimal HbA1c not inclusion criteria; C+, HbA1c is primary outcome; C-, HbA1c is secondary outcome. HbA1c, glycated hemoglobin; SMD, standardized mean difference.

Additional information regarding the case definition of studies included in the meta-analysis is summarized in online supplementary table S3. There were 70 RCTs with data to be pooled, giving a total sample of n=14 796. In the random-effects meta-analysis, while there was a statistically significant reduction in HbA1c for those randomized to a psychological intervention compared with the control group (SMD −0.19, 95% CI −0.25 to −0.12, p<0.001), this was of weak clinical significance, representing an absolute reduction in HbA1c of 3.7 mmol/mol (figure 2). Removal of individual studies had little impact on the overall effect size. There was moderate heterogeneity across studies (I^2^=64.7%, p<0.001) and evidence of publication bias toward positive findings via Egger’s test (p=0.002). No additional studies were considered missing using the trim and fill method.

### Synthesis of results

There was no significant difference in effect size (p=0.12) between interventionist categories when subgroup analyses were conducted for interventions delivered by psychology professionals (n=23, SMD=−0.30, 95% CI −0.46 to –0.14, p<0.001; reduction in HbA1c, 5 mmol/mol), diabetes specialists (n=30, SMD=−0.18, 95% CI −0.25 to –0.10, p<0.001; reduction in HbA1c 3 mmol/mol), and ‘other’ interventionists (n=16, SMD=−0.07, 95% CI −0.21 to 0.06, p=0.29; reduction in HbA1c, 1 mmol/mol). Heterogeneity was high and significant for psychology professionals (I^2^=72.6%, p<0.001) and moderate for diabetes specialists (I^2^=57.7%, p<0.001) and ‘other’ interventionists (I^2^=58.2%, p=0.002). For diabetes specialist delivered studies, there was some evidence of publication bias (p=0.01), but no additional studies were identified as missing using the trim and fill method. For psychology professional and ‘other’ interventionist delivered studies, there was no evidence of publication bias (p=0.09 and p=0.69, respectively), and no additional studies were identified as missing using the trim and fill method.

There was no dose–response association with the number of sessions (b=−0.0063 (95% CI −0.0224 to 0.0097), p=0.43) or duration of psychological intervention (b=−0.06 (95% CI −0.18 to 0.07), p=0.36) or control group (b=−0.02 (95% CI −0.11 to 0.08), p=0.75).

### Additional analyses, non-protocol

We conducted some additional non-protocol analyses. We categorized studies into four groups according to their primary outcome (online supplementary table S4): HbA1c (n=33), psychological (n=19; diabetes empowerment n=1, depressive symptoms n=10, diabetes distress n=4, self-efficacy n=2, stress n=2), self-management behaviors (n=13; physical activity n=6, medication adherence n=5, diet adherence n=2), or biomedical (n=5; coronary heart disease risk n=1, weight n=3, body mass index n=1). There was no association between type of primary outcome and change in HbA1c (p=0.33). A meta-regression revealed no significant difference in effect size in HbA1c reduction between studies where HbA1c was a primary outcome (n=33) compared with studies where HbA1c was a secondary outcome (n=37) (p=0.21).

Out of the 70 included studies, 16 had an inclusion criterion for depressive symptoms, that is, where participants had T2D with comorbid depressive symptoms (figure 2). A meta-regression revealed no significant difference in effect size in HbA1c reduction between studies with comorbid depression inclusion criteria and studies where there were no comorbid depression inclusion criteria (p=0.80). For six of the studies with comorbid depressive symptom inclusion criteria, HbA1c was the primary outcome (online supplementary table S5). A meta-regression was conducted for the interaction between depressive symptoms as an inclusion criterion and whether HbA1c was the study’s primary or secondary outcome; there was no significant difference between the groups (p=0.63). Additionally, some of the comorbid depression studies included collaborative care interventions, and as these could be considered distinct from other psychological interventions we conducted a sensitivity analysis and there was no difference in overall effect size (SMD=−0.19, 95% CI −0.26 to −0.13).

More information regarding inclusion/exclusion criteria of studies included in the meta-analysis can be found in online supplementary table S6.

Eleven studies had an inclusion criterion for suboptimal glycemic control (HbA1c 7.5%/58 mmol/mol or more) (figure 2). A meta-regression revealed no significant difference in effect size in HbA1c reduction between studies where suboptimal HbA1c was an inclusion criterion (SMD=−0.15, 95% CI −0.28 to −0.01) and studies where suboptimal HbA1c was not an inclusion criterion (SMD=−0.19, 95% CI −0.27 to −0.12, p=0.62). For eight studies with suboptimal glycemic control as the inclusion criterion, HbA1c was the primary outcome (online supplementary table S7). A meta-regression was conducted to determine the interaction between studies with suboptimal HbA1c as an inclusion criterion and whether HbA1c was the study’s primary outcome, and there was no significant difference between groups (p=0.51).

### RoB across studies

The RoB domain which was most difficult to assess (ie, coded as ‘unclear RoB’) was the ‘blinding of participants and personnel’ domain (online supplementary figure S2). ‘Selective reporting’ and ‘other bias’ RoB domains were mostly coded as low RoB, while ‘Random sequence generation,’ ‘allocation concealment’ and ‘incomplete outcome data’ showed high RoB across studies.

### Additional analyses: cohort effect

To examine whether there was a cohort effect, we pooled the HbA1c data from 12 RCTs included in an earlier meta-analysis (from inception to January 2003) with the current review (January 2003–July 2018), totaling 82 RCTs (n=15 306). We derived a similar effect size to the current review (SMD −0.20, 95% CI −0.26 to −0.14, p<0.001, equivalent to absolute change in HbA1c of −4 mmol/mol). The effect size was not significantly different between the two meta-analyses (b=−0.13 (95% CI −0.38 to 0.12), p=0.31).

### Additional analyses: NMA

For the NMA there were data available from 70 studies, which included five categories of psychological intervention and three control conditions. In total 146 treatment arms were analyzed (some studies had more than one intervention or control group), with a total sample size of 15 702 (online supplementary table S8). A network plot for all studies demonstrated that 13 out of a possible 28 contrasts could be analyzed (online supplementary figure S3), although to reduce overestimation of treatment effects we only analyzed contrasts with two or more studies. IPT and diabetes education (control) were only studied once and were thus removed from the NMA, including the control group for IPT, resulting in a total number of studies of 142 with a total sample size of 15 573, allowing us to study 11 out of a possible 15 contrasts.

Therefore, direct and indirect effects between CBT, counseling, self-help materials (alternative intervention treatment), usual care, attention control and waiting list control were performed. Online supplementary table S9 shows that the estimated direct and indirect effects between interventions did not differ significantly, with only one exception (counseling vs self-help materials). The non-significant χ^2^ test for inconsistency (χ^2^(8) 8.33, p=0.402, I^2^=3.9%) supports the conclusion of model consistency.

Online supplementary table 10 shows the results of the consistency of NMA comparing all treatments (and controls) against usual care. Self-help materials (this was an additional treatment arm, used in four studies), CBT, and counseling showed a small to moderate treatment effect. Online supplementary table S11 presents the pairwise comparisons of all treatment effects.

**Figure 3:**
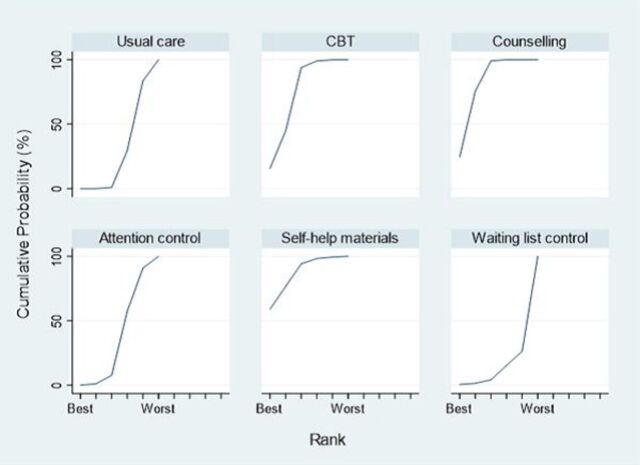
Rankogram for all treatments. The plot shows the surface under the cumulative ranking curves for all treatments for adults with type 2 diabetes. For example, usual care has a very low probability of being among the best treatments but a very high probability of being one of the worst. CBT, cognitive–behavioral therapy.

The rankogram (figure 3) indicated that self-help materials had the highest probability of being the most successful intervention (58.1%), followed by CBT (22.4%) and counseling (18.8%), while waiting list control, attention control and usual care were less likely to be the best treatment (all ≤0.6%). However, an assessment of mean rank and SUCRA suggests little differences between self-help materials, CBT and counseling (online supplementary table S12).

## Conclusions

In this study 94 RCTs were included in the systematic review and 70 had HbA1c data which could be pooled. There was a statistically significant improvement in glycemic control, but this was of weak clinical significance. The absolute reduction in HbA1c of 3.7 mmol/mol is just less than the consensus minimal difference of 4 mmol/mol to reduce the risk of microvascular and cardiovascular disease.^22^ The NMA demonstrated that CBT and counseling interventions were effective compared with controls, but effect sizes were small. Self-help was offered as an alternative treatment to CBT (n=1268) or counseling (n=6105) in four studies and this was effective, but the total sample size was smaller (n=792). There was no difference in the change in glycemic control when interventions were delivered by mental health or non-mental health professionals. Most studies were conducted in North America and Europe.

The strengths of this systematic review were that it was protocolized, registered with PROSPERO (International Prospective Register of Systematic Reviews), conducted according to PRISMA guidelines, and not restricted to English-language publications. We used aggregate and network meta-analysis to optimize the analysis of the pooled data. As we used the same protocol, we were able to link current data with a previous meta-analysis to compare the effects of psychological interventions over 30 years.

The limitations of this review are that by using an older protocol we may have missed some innovative studies, and clinical settings such as multimorbidity and digital interventions where diabetes may have not appeared in the title or abstract. We used outcome data closest to 12-month follow-up as the majority of the included trials were of short duration. We did not review the content of the manuals as there was no systematic method to do so. We included collaborative care interventions under the CBT umbrella, and it could be argued that collaborative care is a complex intervention which differs from other psychological interventions. However, when collaborative care studies^23–25^ were removed from our overall aggregate meta-analysis, there was no significant change in the results. While we were able to determine whether the effect sizes of the current and previous review were different, we were not able to determine if there were differences in effectiveness of psychological interventions according to study population or type of therapy.

Our observation is that in the past 15 years there has been an almost 10-fold increase in the number of RCTs testing the effectiveness of psychological interventions to improve glycemic control as primary or secondary outcome, yet their effectiveness has decreased compared with pre-CONSORT studies. This needs discussion within the diabetes and mental health research and clinical community. Similar patterns of increasing research productivity yet decreasing effectiveness have been observed by others but have not until now been debated.^26–28^ One explanation is that despite guidance on the assessment for fidelity to the psychological intervention,^29^ there is little evidence that this is conducted. Deviations in fidelity to a psychological intervention can lead to dilution of the ‘dose’ and underestimation of its effect. Another possible explanation is the lack of data on the level of proficiency or competency in the delivery of psychological treatments.^29^ A third explanation is whether the primary focus of the psychological treatment is targeting glycemic control or other aspects of diabetes self-management. Only a quarter of studies had links to additional materials or manuals that would give information on the specific content of the intervention. Only a third of the studies were focused on glycemic control. A significant proportion focused on treating depressive symptoms or weight with the secondary outcome that this would improve glycemic control. We also noticed there was no difference in the effect on glycemic control by the profession of the therapist. One interpretation is that diabetes specialists bring diabetes knowledge which is likely to be an important prerequisite to a therapeutic alliance for a person with diabetes. On the other hand, the mental health profession brings psychotherapeutic skills which are also a prerequisite to building a therapeutic relationship. These skills may be more effective when combined. A fifth explanation is that the intervention in the control was of high standard, usual care for diabetes has improved, and the HbA1c national average has dropped in some countries.^2 30^ Last but not least, as the methodological quality has improved with only a handful of RCTs assessed to be of high RoB, another explanation is that collectively these types of interventions, namely CBT and counseling, are not indicated in T2D. The average number of sessions was 7 and the average duration of the intervention was approximately 5 months. T2D is a progressive condition and if a person is not able to make the self-management changes alone or with standard support, it is possible that they are unlikely to do so with a brief relatively inexpensive psychological intervention.

This review highlights a need for a balanced debate. On the one hand there is a clear policy agenda for integrating physical and mental health in diabetes, but there need to be psychological interventions that are effective in improving blood glucose, as ineffective interventions could do more harm and cost health systems more. National and international research strategy led by funding organizations need to invest in innovations in psychological treatments, rather than replicating existing psychological models that are repeatedly delivering very small effect sizes. For instance, there were no studies that used psychodynamic models or addressed the high levels of disordered or addictive eating patterns, stigma of diabetes, or habit formation.^31^

In summary, brief psychological interventions in T2D have limited clinical effectiveness in improving glycemic control.

## References

[1] NICE Type 2 diabetes in adults: management, 2017 Available: https://www.nice.org.uk/guidance/ng28/chapter/1-Recommendations#hba1c-measurement-and-targets [Accessed 26 June 2019].

[2] NDA National diabetes audit, 2016-17, 2018 Retrieved from Available: https://files.digital.nhs.uk/pdf/s/k/national_diabetes_audit_2016-17_report_1__care_processes_and_treatment_targets.pdf [Accessed 24 May 2019].

[3] Anderson RJ, Freedland KE, Clouse RE, et al The prevalence of comorbid depression in adults with diabetes: a meta-analysis. Diabetes Care 2001;24:1069–78. 10.2337/diacare.24.6.1069 11375373

[4] Grigsby AB, Anderson RJ, Freedland KE, et al Prevalence of anxiety in adults with diabetes: a systematic review. J Psychosom Res 2002;53:1053–60. 10.1016/s0022-3999(02)00417-8 12479986

[5] Wild D, von Maltzahn R, Brohan E, et al A critical review of the literature on fear of hypoglycemia in diabetes: implications for diabetes management and patient education. Patient Educ Couns 2007;68:10–15. 10.1016/j.pec.2007.05.003 17582726

[6] Brod M, Kongsø JH, Lessard S, et al Psychological insulin resistance: patient beliefs and implications for diabetes management. Qual Life Res 2009;18:23. 10.1007/s11136-008-9419-1 19039679

[7] Allison KC, Crow SJ, Reeves RR, et al Binge eating disorder and night eating syndrome in adults with type 2 diabetes. Obesity 2007;15:1287–93. 10.1038/oby.2007.150 17495205 PMC2753278

[8] Polonsky WH, Fisher L, Earles J, et al Assessing psychosocial distress in diabetes: development of the diabetes distress scale. Diabetes Care 2005;28:626–31. 10.2337/diacare.28.3.626 15735199

[9] Ismail K, Winkley K, Rabe-Hesketh S Systematic review and meta-analysis of randomised controlled trials of psychological interventions to improve glycaemic control in patients with type 2 diabetes. Lancet 2004;363:1589–97. 10.1016/S0140-6736(04)16202-8 15145632

[10] Moher D, Schulz KF, Altman DG, et al The CONSORT statement: revised recommendations for improving the quality of reports of parallel-group randomised trials. Elsevier, 2001. 11323066

[11] Hoffmann TC, Glasziou PP, Boutron I, et al Better reporting of interventions: template for intervention description and replication (TIDieR) checklist and guide. BMJ 2014;348:g1687. 10.1136/bmj.g1687 24609605

[12] NIHR A systematic review of psychological interventions to improve motivation for self-management in people with type 1 and type 2 diabetes, 2019 Retrieved from Available: https://www.journalslibrary.nihr.ac.uk/programmes/hta/1421310#/ [Accessed 23 May 2019].

[13] Moher D, Liberati A, Tetzlaff J, et al Preferred reporting items for systematic reviews and meta-analyses: the PRISMA statement. Ann Intern Med 2009;151:264–9. 10.7326/0003-4819-151-4-200908180-00135 19622511

[14] Higgins JPT, Altman DG, Gøtzsche PC, et al The Cochrane collaboration's tool for assessing risk of bias in randomised trials. BMJ 2011;343:d5928. 10.1136/bmj.d5928 22008217 PMC3196245

[15] Egger M, Davey Smith G, Schneider M, et al Bias in meta-analysis detected by a simple, graphical test. BMJ 1997;315:629–34. 10.1136/bmj.315.7109.629 9310563 PMC2127453

[16] Duval S Tweedie R. A nonparametric “trim and fill” method of accounting for publication bias in meta-analysis. J Am Stat Assoc 2000;95:89–98.

[17] Borenstein LH M, Higgins JPT, Rothstein HR Introduction to meta-analysis, 2011.

[18] Riley RD, Jackson D, Salanti G, et al Multivariate and network meta-analysis of multiple outcomes and multiple treatments: rationale, concepts, and examples. BMJ 2017;358:j3932. 10.1136/bmj.j3932 28903924 PMC5596393

[19] White IR, Barrett JK, Jackson D, et al Consistency and inconsistency in network meta-analysis: model estimation using multivariate meta-regression. Res Synth Methods 2012;3:111–25. 10.1002/jrsm.1045 26062085 PMC4433771

[20] Higgins JPT, Jackson D, Barrett JK, et al Consistency and inconsistency in network meta-analysis: concepts and models for multi-arm studies. Res Synth Methods 2012;3:98–110. 10.1002/jrsm.1044 26062084 PMC4433772

[21] White IR, Thomas J Standardized mean differences in individually-randomized and cluster-randomized trials, with applications to meta-analysis. Clin Trials 2005;2:141–51. 10.1191/1740774505cn081oa 16279136

[22] Baxter M, Hudson R, Mahon J, et al Estimating the impact of better management of glycaemic control in adults with type 1 and type 2 diabetes on the number of clinical complications and the associated financial benefit. Diabet Med 2016;33:1575–81. 10.1111/dme.13062 26773733

[23] Chwastiak LA, Luongo M, Russo J, et al Use of a mental health center collaborative care team to improve diabetes care and outcomes for patients with psychosis. Psychiatr Serv 2018;69:349–52. 10.1176/appi.ps.201700153 29191136

[24] Ell K, Katon W, Xie B, et al One-Year postcollaborative depression care trial outcomes among predominantly Hispanic diabetes safety net patients. Gen Hosp Psychiatry 2011;33:436–42. 10.1016/j.genhosppsych.2011.05.018 21774987 PMC3175272

[25] Williams JW, Katon W, Lin EHB, et al The effectiveness of depression care management on diabetes-related outcomes in older patients. Ann Intern Med 2004;140:1015–24. 10.7326/0003-4819-140-12-200406150-00012 15197019

[26] Ekong G, Kavookjian J Motivational interviewing and outcomes in adults with type 2 diabetes: a systematic review. Patient Educ Couns 2016;99:944–52. 10.1016/j.pec.2015.11.022 26699083

[27] Pillay J, Armstrong MJ, Butalia S, et al Behavioral programs for type 2 diabetes mellitus: a systematic review and network meta-analysis. Ann Intern Med 2015;163:848–60. 10.7326/M15-1400 26414227

[28] Xie J, Deng W Psychosocial intervention for patients with type 2 diabetes mellitus and comorbid depression: a meta-analysis of randomized controlled trials. Neuropsychiatr Dis Treat 2017;13:2681–90. 10.2147/NDT.S116465 29123401 PMC5661466

[29] Gearing RE, El-Bassel N, Ghesquiere A, et al Major ingredients of fidelity: a review and scientific guide to improving quality of intervention research implementation. Clin Psychol Rev 2011;31:79–88. 10.1016/j.cpr.2010.09.007 21130938

[30] Ali MK, Bullard KM, Saaddine JB, et al Achievement of goals in U.S. diabetes care, 1999-2010. N Engl J Med 2013;368:1613–24. 10.1056/NEJMsa1213829 23614587

[31] Gardner B, Rebar AL Habit formation and behavior change. Oxford Research Encyclopedia of Psychology, 2019.

