# Supplementary material for: Psychological interventions to improve glycemic control in adults with type 2 diabetes: a systematic review and meta-analysis
Source: BMJ Open Diabetes Res Care. 2020 Apr 8;8(1):e001150. doi: 10.1136/bmjdrc-2019-001150 (PMC7254106; doi:10.1136/bmjdrc-2019-001150)
Supplement: Supplementary data [file bmjdrc-2019-001150supp001.pdf]

Supplementary reference list

S1. Whittemore R, Melkus GD, Sullivan A, Grey M. A nurse-coaching intervention for women with type 2 diabetes. Diabetes Educator. 2004;30(5):795-804.

S2. Williams JW, Katon W, Lin EH, Nöel PH, Worchel J, Cornell J, et al. The effectiveness of depression care management on diabetes-related outcomes in older patients. Annals of internal medicine. 2004;140(12):1015-24.

S3. Siebolds M, Gaedeke O, Schwedes U. Self-monitoring of blood glucose--Psychological aspects relevant to changes in HbA1c in type 2 diabetic patients treated with diet or diet plus oral antidiabetic medication. Patient Education and Counseling. 2006;62(1):104-10.

S4. Keeratiyutawong P, Hanucharurnkul S, Melkus GDE, Panpakdee O, Vorapongsathorn T. Effectiveness of a self-management program for Thais with type 2 diabetes. Thai Journal of Nursing Research. 2006;10(2):85-97 13p.

S5. Gregg JA, Callaghan GA, Hayes SC, Glenn-Lawson JL. Improving diabetes self-management through acceptance, mindfulness, and values: A randomized controlled trial. Journal of Consulting and Clinical Psychology. 2007;75(2):336-43.

S6. West DS, DiLillo V, Bursac Z, Gore SA, Greene PG. Motivational interviewing improves weight loss in women with type 2 diabetes. Diabetes Care. 2007;30(5):1081-7.

S7. Dale J, Caramlau I, Sturt J, Friede T, Walker R. Telephone peer-delivered intervention for diabetes motivation and support: the telecare exploratory RCT. Patient education and counseling. 2009;75(1):91-8.

S8. Davazdah Emamy M, Roshan R, Mehrabi A, Attari A. The effectiveness of cognitive-behavioral stress management training on glycemic control and depression in patients with type 2 diabetes. Iranian Journal of Endocrinology and Metabolism. 2009;11(4):385-92.

S9. Sacco WP, Malone, J. I., Morrison, A. D., Friedman, A., & Wells, K. Effect of a brief, regular telephone intervention by paraprofessionals for type 2 diabetes. Journal of behavioral medicine. 2009;32(4).

S10. Evans G, Lewin TJ, Bowen K, Lowe J. Dealing with anxiety: A pilot cognitive behavioural therapy program for diabetic clinic outpatient attendees. International Journal of Diabetes Mellitus. 2010;2(1):51-5.

S11. Wolever R, Dreusicke M, Fikkan J, Hawkins T, Yeung S, Wakefield J, et al. Integrative health coaching for patients with type 2 diabetes: A randomized clinical trial. The Diabetes Educator. 2010;36(4):629-39.

S12. D'Eramo Melkus G, Chyun D, Vorderstrasse A, Newlin K, Jefferson V, Langerman S. The effect of a diabetes education, coping skills training, and care intervention on physiological and psychosocial outcomes in black women with type 2 diabetes. Biological Research for Nursing. 2010;12(1):7-19.

S13. De Greef K, Deforche B, Tudor-Locke C, De Bourdeaudhuij I. A cognitive-behavioural pedometer-based group intervention on physical activity and sedentary behaviour in individuals with type 2 diabetes. Health Education Research. 2010;25(5):724-36.

S14. Hawkins SY. Improving glycemic control in older adults using a videophone motivational diabetes self-management intervention. Research & Theory for Nursing Practice. 2010;24(4):217-32.

S15. Osborn CY, Amico K, Cruz N, O'Connell AA, Perez-Escamilla R, Kalichman SC, et al. A brief culturally tailored intervention for Puerto Ricans with type 2 diabetes. Health Education & Behavior. 2010;37(6):849-62 14p.

S16. Garcia-Huidobro D, Bittner M, Brahm P, Puschel K. Family intervention to control type 2 diabetes: a controlled clinical trial. Family Practice. 2011;28(1):4-11.

S17. Keogh KM, Smith SM, White P, McGilloway S, Kelly A, Gibney J, et al. Psychological family intervention for poorly controlled type 2 diabetes. American Journal of Managed Care. 2011;17(2):105-13.

S18. De Greef K, Deforche B, Tudor-Locke C, De Bourdeaudhuij I. Increasing Physical Activity in Belgian Type 2 Diabetes Patients: a Three-Arm Randomized Controlled Trial. International Journal of Behavioral Medicine. 2011;18(3):188-98.
S19. Hamid N. Effects of stress management training on glycemic control in women with type 2 diabetes. 2011.
S20. Piette JD, Richardson C, Himle J, Duffy S, Torres T, Vogel M, et al. A randomized trial of telephonic counseling plus walking for depressed diabetes patients. Medical Care. 2011;49(7):641-8.
S21. Lamers F, Jonkers CC, Bosma H, Knottnerus J, van Eijk JT. Treating depression in diabetes patients: Does a nurse-administered minimal psychological intervention affect diabetes-specific quality of life and glycaemic control? A randomized controlled trial. Journal of Advanced Nursing. 2011;67(4):788-99.
S22. Welch G, Zagarins SE, Feinberg RG, Garb JL. Motivational interviewing delivered by diabetes educators: does it improve blood glucose control among poorly controlled type 2 diabetes patients? Diabetes Research & Clinical Practice. 2011;91(1):54-60.
S23. Ell K, Katon W, Xie B, Lee P-J, Kapetanovic S, Guterman J, et al. One-year postcollaborative depression care trial outcomes among predominantly Hispanic diabetes safety net patients. General hospital psychiatry. 2011;33(5):436-42.
S24. Farmer A, Hardeman W, Hughes D, Prevost AT, Kim Y, Craven A, et al. An explanatory randomised controlled trial of a nurse-led, consultation-based intervention to support patients with adherence to taking glucose lowering medication for type 2 diabetes. BMC family practice. 2012;13:30.
S25. Penckofer SM, Ferrans C, Mumby P, Byrn M, Emanuele MA, Harrison PR, et al. A psychoeducational intervention (SWEEP) for depressed women with diabetes. Annals of Behavioral Medicine. 2012;44(2):192-206.
S26. Hartmann M, Kopf S, Kircher C, Faude-Lang V, Djuric Z, Augstein F, et al. Sustained effects of a mindfulness-based stress-reduction intervention in type 2 diabetic patients: design and first results of a randomized controlled trial (the Heidelberger Diabetes and Stress-study). Diabetes Care. 2012;35(5):945-7.
S27. Chen SM, Creedy D, Lin H-S, Wollin J. Effects of motivational interviewing intervention on self-management, psychological and glycemic outcomes in type 2 diabetes: a randomized controlled trial. International journal of nursing studies. 2012;49(6):637-44.
S28. Plotnikoff RC, Karunamuni N, Courneya KS, Sigal RJ, Johnson JA, Johnson ST. The Alberta Diabetes and Physical Activity Trial (ADAPT): a randomized trial evaluating theory-based interventions to increase physical activity in adults with type 2 diabetes. Annals of Behavioral Medicine. 2013;45(1):45-56.
S29. Welschen LM, van Oppen P, Bot SD, Kostense PJ, Dekker JM, Nijpels G. Effects of a cognitive behavioural treatment in patients with type 2 diabetes when added to managed care; a randomised controlled trial. Journal of behavioral medicine. 2013;36(6):556-66.
S30. Mandel SE, Davis BA, Secic M. Effects of music therapy and music-assisted relaxation and imagery on health-related outcomes in diabetes education: a feasibility study. Diabetes Educator. 2013;39(4):568-81.
S31. Jansink R, Braspenning J, Keizer E, van der Weijden T, Elwyn G, Grol R. No identifiable Hb1Ac or lifestyle change after a comprehensive diabetes programme including motivational interviewing: a cluster randomised trial. Scandinavian journal of primary health care. 2013;31(2):119-27.
S32. Juul L, Maindal HT, Zoffmann V, Frydenberg M, Sandbaek A. Effectiveness of a training course for general practice nurses in motivation support in type 2 diabetes care: a cluster-randomised trial. PLoS ONE [Electronic Resource]. 2014;9(5):e96683.
S33. Steed L, Barnard M, Hurel S, Jenkins C, Newman S. How does change occur following a theoretically based self-management intervention for type 2 diabetes. Psychology Health & Medicine. 2014;19(5):536-46.

S34. Safren SA, Gonzalez JS, Wexler DJ, Psaros C, Delahanty LM, Blashill AJ, et al. A randomized controlled trial of cognitive behavioral therapy for adherence and depression (CBT-AD) in patients with uncontrolled type 2 diabetes. Diabetes Care. 2014;37(3):625-33.
S35. Gois C, Dias V, Carmo I, Duarte R, Ferro A, Santos A, et al. Treatment response in type 2 diabetes patients with major depression. Clinical Psychology & Psychotherapy. 2014;21(1):39-48.
S36. Li M, Li T, Shi B-Y, Gao C-X. Impact of motivational interviewing on the quality of life and its related factors in type 2 diabetes mellitus patients with poor long-term glycemic control. International journal of nursing sciences. 2014;1(3):250-4.
S37. Griffin SJ, Simmons RK, Prevost AT, Williams KM, Hardeman W, Sutton S, et al. Multiple behaviour change intervention and outcomes in recently diagnosed type 2 diabetes: The ADDITION-Plus randomised controlled trial. Diabetologia. 2014;57(7):1308-19.
S38. Eakin EG, Winkler EA, Dunstan DW, Healy GN, Owen N, Marshall AM, et al. Living well with diabetes: 24-month outcomes from a randomized trial of telephone-delivered weight loss and physical activity intervention to improve glycemic control. Diabetes Care. 2014;37(8):2177-85.
S39. van Son J, Nyklíček I, Pop VJ, Blonk MC, Erdtsieck RJ, Pouwer F. Mindfulness-based cognitive therapy for people with diabetes and emotional problems: long-term follow-up findings from the DiaMind randomized controlled trial. Journal of psychosomatic Research. 2014;77(1):81-4.
S40. Kim MT, Kim KB, Huh B, Nguyen T, Han HR, Bone LR, et al. The Effect of a Community-Based Self-Help Intervention: Korean Americans With Type 2 Diabetes. American Journal of Preventive Medicine. 2015;49(5):726-37.
S41. Chlebowy DO, El-Mallakh P, Myers J, Kubiak N, Cloud R, Wall MP. Motivational Interviewing to Improve Diabetes Outcomes in African Americans Adults With Diabetes. Western Journal of Nursing Research. 2015;37(5):566-80.
S42. Pladevall M, Divine G, Wells KE, Resnicow K, Williams L. A randomized controlled trial to provide adherence information and motivational interviewing to improve diabetes and lipid control. The Diabetes Educator. 2015;41(1):136-46.
S43. Hermanns N, Schmitt A, Gahr A, Herder C, Nowotny B, Roden M, et al. The effect of a Diabetes-Specific Cognitive Behavioral Treatment Program (DIAMOS) for patients with diabetes and subclinical depression: results of a randomized controlled trial. Diabetes Care. 2015;38(4):551-60.
S44. Pibernik-Okanović M, Hermanns, N., Ajduković, D., Kos, J., Prašek, M., Šekerija, M., & Lovrenčić, M. V. . Does treatment of subsyndromal depression improve depression-related and diabetes-related outcomes? A randomised controlled comparison of psychoeducation, physical exercise and enhanced treatment as usual. Trials. 2015;16(1):305.
S45. Petrak F, Herpertz, S., Albus, C., Hermanns, N., Hiemke, C., Hiller, W., ... & Zahn, D. Cognitive Behavioral Therapy Versus Sertraline in Patients With Depression and Poorly Controlled Diabetes: The Diabetes and Depression (DAD) Study A Randomized Controlled Multicenter Trial. Diabetes care. 2015;38(5):767-75.
S46. Huang CY, Lai HL, Chen CI, Lu YC, Li SC, Wang LW, et al. Effects of motivational enhancement therapy plus cognitive behaviour therapy on depressive symptoms and health-related quality of life in adults with type II diabetes mellitus: a randomised controlled trial. Quality of Life Research. 2016;25(5):1275-83.
S47. Browning C, Chapman A, Yang H, Liu S, Zhang TH, Enticott JC, et al. Management of type 2 diabetes in China: the Happy Life Club, a pragmatic cluster randomised controlled trial using health coaches. Bmj Open. 2016;6(3).
S48. Kasteleyn MJ, Vos, R. C., Rijken, M., Schellevis, F. G., & Rutten, G. E. H. M. Effectiveness of tailored support for people with Type 2 diabetes after a first acute coronary event: a multicentre randomized controlled trial (the Diacourse-ACE study). Diabetic Medicine. 2016;33(1):125-33.
S49. Chiu, Y-HH, LAW, EAB, Y-CY, J-SW, et al. Dissemination of evidence-base minimal psychological intervention for diabetes management in Taiwan adults with type 2 diabetes. International Journal of Clinical and Experimental Medicine. 2016;9(7):14489-98.

S50. Fan M-H, Huang B-T, Tang Y-C, Han X-H, Dong W-W, Wang L-X. Effect of individualized diabetes education for type 2 diabetes mellitus: a single-center randomized clinical trial. African health sciences. 2016;16(4):1157-62.
S51. Juul L, Andersen VJ, Arnoldsen J, Maindal HT. Effectiveness of a brief theory-based health promotion intervention among adults at high risk of type 2 diabetes: One-year results from a randomised trial in a community setting. Primary care diabetes. 2016;10(2):111-20.
S52. Shayeghian Z, Hassanabadi H, Aguilar-Vafaie ME, Amiri P, Besharat MA. A randomized controlled trial of acceptance and commitment therapy for type 2 diabetes management: the moderating role of coping styles. PloS one. 2016;11(12):e0166599.
S53. Wagner JA, Bermudez-Millan A, Damio G, Segura-Perez S, Chhabra J, Vergara C, et al. A randomized, controlled trial of a stress management intervention for Latinos with type 2 diabetes delivered by community health workers: outcomes for psychological wellbeing, glycemic control, and cortisol. Diabetes research and clinical practice. 2016;120:162-70.
S54. Akturan S, Kaya ÇA, Ünalan PC, Akman M. The effect of the BATHE interview technique on the empowerment of diabetic patients in primary care: a cluster randomised controlled study. Primary care diabetes. 2017;11(2):154-61.
S55. Balducci S, D'Errico V, Haxhi J, Sacchetti M, Orlando G, Cardelli P, et al. Effect of a behavioral intervention strategy for adoption and maintenance of a physically active lifestyle: the Italian diabetes and exercise study 2 (IDES_2): a randomized controlled trial. Diabetes care. 2017;40(11):1444-52.
S56. Chee WS, Singh HKG, Hamdy O, Mechanick JI, Lee VK, Barua A, et al. Structured lifestyle intervention based on a trans-cultural diabetes-specific nutrition algorithm (tDNA) in individuals with type 2 diabetes: a randomized controlled trial. BMJ Open Diabetes Research and Care. 2017;5(1):e000384.
S57. Egede LE, Walker RJ, Payne EH, Knapp RG, Acierno R, Frueh BC. Effect of psychotherapy for depression via home telehealth on glycemic control in adults with type 2 diabetes: Subgroup analysis of a randomized clinical trial. Journal of telemedicine and telecare. 2018;24(9):596-602.
S58. Furler J, O'Neal D, Speight J, Manski-Nankervis J-A, Gorelik A, Holmes-Truscott E, et al. Supporting insulin initiation in type 2 diabetes in primary care: results of the Stepping Up pragmatic cluster randomised controlled clinical trial. bmj. 2017;356:j783.
S59. Hermanns N, Ehrmann D, Schall S, Maier B, Haak T, Kulzer B. The effect of an education programme (MEDIAS 2 BSC) of non-intensive insulin treatment regimens for people with Type 2 diabetes: a randomized, multi-centre trial. Diabetic Medicine. 2017;34(8):1084-91.
S60. Muñoz-Flórez A, Cortés O. Impacto de la Entrevista Motivacional en la Adherencia de Pacientes Diabéticos Inactivos a la Actividad Física: Estudio Piloto de un Ensayo Clínico EMOACTIF–DM. Revista Colombiana de Psicología. 2017;26(2):263-81.
S61. Rees G, O'Hare F, Saeed M, Sudholz B, Sturrock BA, Xie J, et al. Problem-solving therapy for adults with diabetic retinopathy and diabetes-specific distress: a pilot randomized controlled trial. BMJ Open Diabetes Research and Care. 2017;5(1):e000307.
S62. Carrasquillo O, Lebron C, Alonzo Y, Li H, Chang A, Kenya S. Effect of a community health worker intervention among Latinos with poorly controlled type 2 diabetes: the Miami Healthy Heart Initiative randomized clinical trial. JAMA internal medicine. 2017;177(7):948-54.
S63. Gomes LC, Coelho ACM, dos Santos Gomides D, Foss-Freitas MC, Foss MC, Pace AE. Contribution of family social support to the metabolic control of people with diabetes mellitus: a randomized controlled clinical trial. Applied Nursing Research. 2017;36:68-76.
S64. Jiang X, Fan X, Wu R, Geng F, Hu C. The effect of care intervention for obese patients with type II diabetes. Medicine. 2017;96(42).
S65. Chew B-H, Vos RC, Stellato RK, Ismail M, Rutten GE. The effectiveness of an emotion-focused educational programme in reducing diabetes distress in adults with Type 2 diabetes mellitus (VEMOFIT): a cluster randomized controlled trial. Diabetic Medicine. 2018;35(6):750-9.

S66. Chwastiak LA, Luongo M, Russo J, Johnson L, Lowe JM, Hoffman G, et al. Use of a Mental Health Center Collaborative Care Team to Improve Diabetes Care and Outcomes for Patients With Psychosis. Psychiatric Services. 2017;69(3):349-52.
S67. Döbler A, Herbeck Belnap B, Pollmann H, Farin E, Raspe H, Mittag O. Telephone-delivered lifestyle support with action planning and motivational interviewing techniques to improve rehabilitation outcomes. Rehabilitation psychology. 2018;63(2):170.
S68. Ismail K, Winkley K, de Zoysa N, Patel A, Heslin M, Graves H, et al. Nurse-led psychological intervention for type 2 diabetes: a cluster randomised controlled trial (Diabetes-6 study) in primary care. The British journal of general practice : the journal of the Royal College of General Practitioners. 2018.
S69. Momtazi S, Salimi C, Zenouzian S, Shourani MJ, Urquhart C. Motivational Interviewing as Group Therapy for Glycemic Control and Treatment Satisfaction of Patients with Type 2 Diabetes Mellitus. Middle East Journal of Family Medicine. 2018;7(10):75.
S70. Wroe AL, Rennie EW, Sollesse S, Chapman J, Hassy A. Is cognitive behavioural therapy focusing on depression and anxiety effective for people with long-term physical health conditions? A controlled trial in the context of type 2 diabetes mellitus. Behavioural and cognitive psychotherapy. 2018;46(2):129-47.
S71. Clark M, Hampson SE, Avery L, Simpson R. Effects of a tailored lifestyle self-management intervention in patients with Type 2 diabetes. British journal of health psychology. 2004;9(3):365-79.
S72. Karlsen B, Idsoe T, Dirdal I, Rokne Hanestad B, Bru E. Effects of a group-based counselling programme on diabetes-related stress, coping, psychological well-being and metabolic control in adults with type 1 or type 2 diabetes. Patient education and counseling. 2004;53(3):299-308.
S73. Hokanson JM, Anderson RL, Hennrikus DJ, Lando HA, Kendall DM. Integrated tobacco cessation counseling in a diabetes self-management training program: a randomized trial of diabetes and reduction of tobacco. Diabetes Educator. 2006;32(4):562-70.
S74. Heinrich E, Candel M, Schaper NC, de Vries NK. Effect evaluation of a Motivational Interviewing based counselling strategy in diabetes care. Diabetes Research and Clinical Practice. 2010;90(3):270-8.
S75. Pourisharif H, Babapour J, Zamani R, Besharat MA, Mehryar AH, Rajab A. The effectiveness of motivational interviewing in improving health outcomes in adults with type 2 diabetes. In: Hacifazlioglu O, Halat MM, editors. Wcpcg 2010. Procedia Social and Behavioral Sciences. 52010. p. 1580-4.
S76. Castelnuovo G, Manzoni GM, Cuzziol P, Cesa GL, Corti S, Tuzzi C, et al. TECNOB Study: Ad Interim Results of a Randomized Controlled Trial of a Multidisciplinary Telecare Intervention for Obese Patients with Type-2 Diabetes. Clinical Practice & Epidemiology in Mental Health [Electronic Resource]: CP & EMH. 2011;7:44-50.
S77. Waker CL. The Effects of Motivational Interviewing on Diabetes Self-Management Behaviors and Glycemic Control in Type 2 Diabetes: A Translational Study: University of Cincinnati; 2012.
S78. Gabbay RA, Añel-Tiangco RM, Dellasega C, Mauger DT, Adelman A, Van Horn DH. Diabetes nurse case management and motivational interviewing for change (DYNAMIC): Results of a 2-year randomized controlled pragmatic trial. Journal of diabetes. 2013;5(3):349-57.
S79. Inouye J, Li D, Davis J, Arakaki R. Psychosocial and Clinical Outcomes of a Cognitive Behavioral Therapy for Asians and Pacific Islanders with Type 2 Diabetes: A Randomized Clinical Trial. Hawai'i Journal of Medicine & Public Health : A Journal of Asia Pacific Medicine & Public Health. 2015;74(11):360-8.
S80. Fitzpatrick SL, Golden, S. H., Stewart, K., Sutherland, J., DeGross, S., Brown, T., ... & Hill-Briggs, F. Effect of DECIDE (Decision-making Education for Choices In Diabetes Everyday) Program Delivery Modalities on Clinical and Behavioral Outcomes in Urban African Americans With Type 2 Diabetes: A Randomized Trial. . Diabetes Care. 2016;39:2149–57.

S81. Rondags SM, de Wit M, Twisk JW, Snoek FJ. Effectiveness of HypoAware, a brief partly web-based psychoeducational intervention for adults with type 1 and insulin-treated type 2 diabetes and problematic hypoglycemia: a cluster randomized controlled trial. Diabetes Care. 2016;39(12):2190-6.
S82. Cummings DM, Lutes LD, Littlewood K, Solar C, Hambidge B, Gatlin P. Impact of distress reduction on behavioral correlates and A1C in African American women with uncontrolled type 2 diabetes: Results from EMPOWER. Ethnicity & disease. 2017;27(2):155.
S83. Egede LE, Williams JS, Voronca DC, Gebregziabher M, Lynch CP. Telephone-delivered behavioral skills intervention for African American adults with type 2 diabetes: a randomized controlled Trial. Journal of general internal medicine. 2017;32(7):775-82.
S84. Friis AM, Johnson MH, Cutfield RG, Consedine NS. Kindness matters: a randomized controlled trial of a mindful self-compassion intervention improves depression, distress, and HbA1c among patients with diabetes. Diabetes care. 2016;39(11):1963-71.
S85. Tovote KA, Schroevers MJ, Snippe E, Emmelkamp PM, Links TP, Sanderman R, et al. What works best for whom? Cognitive Behavior Therapy and Mindfulness-Based Cognitive Therapy for depressive symptoms in patients with diabetes. PloS one. 2017;12(6):e0179941.
S86. Whitehead LC, Crowe MT, Carter JD, Maskill VR, Carlyle D, Bugge C, et al. A nurse-led education and cognitive behaviour therapy-based intervention among adults with uncontrolled type 2 diabetes: A randomised controlled trial. Journal of evaluation in clinical practice. 2017;23(4):821-9.
S87. Berk KA, Buijks HI, Verhoeven AJ, Mulder MT, Özcan B, van't Spijker A, et al. Group cognitive behavioural therapy and weight regain after diet in type 2 diabetes: results from the randomised controlled POWER trial. Diabetologia. 2018;61(4):790-9.
S88. Armani Kian A, Vahdani B, Noorbala A, Nejatisafa A, Arbabi M, Zenoozian S, et al. The Impact of Mindfulness-Based Stress Reduction on Emotional Wellbeing and Glycemic Control of Patients with Type 2 Diabetes Mellitus. Journal of diabetes research. 2018;2018.
S89. Pyatak EA, Carandang K, Vigen CL, Blanchard J, Diaz J, Concha-Chavez A, et al. Occupational therapy intervention improves glycemic control and quality of life among young adults with diabetes: the resilient, empowered, active living with diabetes (REAL Diabetes) randomized controlled trial. Diabetes care. 2018;41(4):696-704.
S90. Lin EH, Von Korff M, Peterson D, Ludman EJ, Ciechanowski P, Katon W. Population targeting and durability of multimorbidity collaborative care management. American Journal of Managed Care. 2014;20(11):887-95.
S91. Minet LKR, Wagner L, Lonvig EM, Hjelmborg J, Henriksen JE. The effect of motivational interviewing on glycaemic control and perceived competence of diabetes self-management in patients with type 1 and type 2 diabetes mellitus after attending a group education programme: a randomised controlled trial. Diabetologia. 2011;54(7):1620-9.
S92. Safford MM, Andreae S, Cherrington AL, Martin MY, Halanych J, Lewis M, et al. Peer Coaches to Improve Diabetes Outcomes in Rural Alabama: A Cluster Randomized Trial. Annals of Family Medicine. 2015;13 Suppl 1:S18-26.
S93. Schroevers MJ, Tovote K, Keers JC, Links TP, Sanderman R, Fleer J. Individual mindfulness-based cognitive therapy for people with diabetes: A pilot randomized controlled trial. Mindfulness. 2015;6(1):99-110.
S94. Weinger K, Beverly EA, Lee Y, Sitnokov L, Ganda OP, Caballero AE. The effect of a structured behavioral intervention on poorly controlled diabetes: a randomized controlled trial. Archives of Internal Medicine. 2011;171(22):1990-9.
