# Supplementary material for: Psychological interventions to improve glycemic control in adults with type 2 diabetes: a systematic review and meta-analysis
Source: BMJ Open Diabetes Res Care. 2020 Apr 8;8(1):e001150. doi: 10.1136/bmjdrc-2019-001150 (PMC7254106; doi:10.1136/bmjdrc-2019-001150)
Supplement: Supplementary data [file bmjdrc-2019-001150supp002.pdf]

Appendix 1: Supplementary Online Material

Results

Table S1 – Search strategy for the systematic review of psychological interventions for people with Type 2 diabetes

1. exp Diabetes Mellitus/
2. diabet$.ab,ti.
3. (DKA or IDDM).mp. or DMI.ab,ti. [mp=title, original title, abstract, name of substance word, subject heading word]
4. (MODY or DM2 or NIDDM).mp. or IIDM.ti,ab. [mp=title, original title, abstract, name of substance word, subject heading word]
5. insulin$ secret$ dysfunc$.ti,ab.
6. insulin$ resist$.ti,ab.
7. ((impaired glucose tolerance or glucose intoleran$ or insulin$ resist$) and (DM or DM2)).ti,ab.
8. insulin$ depend$.mp. or insulin?depend$.ti,ab. [mp=title, original title, abstract, name of substance word, subject heading word]
9. (non insulin$ depend$ or nonisulin$ depend$ or nonisulin?depend).mp. or non insulin?depend$.ti,ab. [mp=title, original title, abstract, name of substance word, subject heading word]
10. (("typ$ 1" or typ$ I) adj6 DM).ti,ab.
11. (("typ$ 2" or typ$ II) adj6 DM).ti,ab.
12. ((juvenil$ or child$ or keto$ or labil$ or brittl$ or earl$ onset) adj6 (DM or DM1)).ti,ab.
13. ((keto$ prone or autoimmun$ or auto immun$ or sudden onset) adj6 (DM or DM1)).ti,ab.
14. ((keto$ resist$ or nonketo$ or non keto$ or adult$ onset or matur$ onset or late$ onset or slow onset or stabl$) adj6 (DM or DM2)).ti,ab.
15. exp Insulin Resistance/
16. (insulin$ defic$ adj6 (absolut$ or relativ$)).ti,ab.
17. metabolic$ syndrom$.ti,ab.
18. (syndrom$ X not (fragil$ X or X linked)).ti,ab.
19. (plurimetabolic$ syndrom$ or pluri metabolic$ syndrom$).ti,ab.
20. or/1-19
21. exp Psychotherapy/
22. exp Counseling/
23. exp Mood disorders/
24. exp Depression/
25. psycho$.mp
26. counsel$.mp
27. depression.mp
28. depressive.mp
29. (interpersonal adj5 therap$).mp
30. art therap$.mp
31. aversion therap$.mp
32. balint.mp
33. behavio?r adj5 (intervention or therap* or modific*)
34. cognitive adj5 (therap* or intervention or program* or train* or theory)
35. (family adj3 (intervention or treatment or counsel* or therap*)
36. colo?r therap$.mp.
37. crisis intervention.mp
38. dance therap$.mp

Winkley K, *et al. BMJ Open Diab Res Care* 2020; 8:e001150. doi: 10.1136/bmjdrc-2019-001150

39. gestalt therap$.mp
40. music therap$.mp
41. milieu therap$.mp
42. (assert$ adj5 training).mp
43. Narrative therap$.mp.
44. nondirective therap$.mp
45. (problem solving adj5 therap$).mp
46. (self control adj5 therap$).mp
47. person cent$.mp
48. client cent$.mp
49. psychodrama$.mp
50. paradoxical technique$.mp
51. play therap$.mp
52. rational emotive.mp
53. reality therap$.mp
54. role play$.mp
55. (relax$ adj5 training).mp
56. sociotherap$.mp
57. socioenvironmental.mp
58. supportive therap$.mp
59. transactional.mp
60. acceptance adj2 (commitment therap*)
61. coping skills training.mp.
62. exp Mindfulness/
63. motivation* adj2 (interview* or therap*)
64. multisystemic therapy
65. or/21-64
66. Randomized Controlled Trials as Topic/
67. randomized controlled trial/
68. Random Allocation/
69. Double Blind Method/
70. Single Blind Method/
71. clinical trial/
72. clinical trial, phase i.pt
73. clinical trial, phase ii.pt
74. clinical trial, phase iii.pt
75. clinical trial, phase iv.pt
76. controlled clinical trial.pt
77. randomized controlled trial.pt
78. multicenter study.pt
79. clinical trial.pt
80. exp Clinical Trials as topic/
81. (clinical adj25 trial$).tw
82. ((singl$ or doubl$ or treb$ or tripl$) adj25 (blind$3 or mask$3)).tw
83. PLACEBOS/
84. placebo$.tw
85. randomly allocated.tw
86. (allocated adj2 random$).tw
87. Or/66-86
88. case report.tw
89. letter/

90. historical article/
91. Or/ 88-90
92. 87 NOT 91
93. 20 AND 65 AND 92
94. limit 88 to yr="2003 -Current"

Winkley K, *et al. BMJ Open Diab Res Care* 2020; 8:e001150. doi: 10.1136/bmjdrc-2019-001150

Table S2- Study and intervention characteristics of RCTs

Year, Country, reference	Total number of participants	Type of psychological intervention	Number of sessions in intervention	Intervention Description (Intervention name, facilitator, format, individual/group)	Control Description (Control category, facilitator, format, individual/group)
				**Studies included in meta-analysis and systematic review**	
2004, USA, Whittemore [S1]	49	Counselling	6	Nurse-Coaching Intervention, Nurses, face-to-face, individual.	Usual care
2004, USA, Williams [S2]	293	CBT	6-8	Collaborative care (depression treatment including problem solving treatment); depression clinical specialist + GP; face-to-face; individual	Usual care
2006, Germany, Siebolds [S3]	223	Counselling	4	Counselling, Physician, face-to-face, individual.	Dietary counselling, Physician, face-to-face, individual.
2006, Thailand, Keeratiyutawong [S4]	90	CBT	5	Self-management Group; Psychology researcher; face-to face; group	Diabetes education; Diabetes health care team; face-to-face; individual
2007, USA, Gregg [S5]	81	CBT	1	Acceptance and commitment therapy (ACT), Psychologist, face-to-face, group.	Diabetes education, psychology masters-level students, face-to-face, group.
2007, USA, West [S6]	217	Counselling	5	Motivational interviewing; clinical psychologists; face-to-face; individual	Diabetes education; health educators; face-to-face; individual
2009, UK, Dale [S7]	231	Counselling	6	1) Telephone support (motivational interviewing); nurses; telephone; individual<br>2) Telephone support (motivational interviewing); peers; telephone; individual	Usual care
2009, Iran, Davazdah [S8]	40	CBT	12	CBT, trained researcher, face to face, Group	Waiting list; see intervention description
2009, USA, Sacco [S9]	62	Counselling	18	Telephone ‘‘coaching’’ intervention; Undergraduates in Psychology; telephone; individual	Usual care
2010, Australia, Evans [S10]	60	CBT	7	CBT; face to face; group	Waiting list (usual care for 3 months then intervention)
2010, USA, Wolever [S11]	56	Counselling	14	Integrative health (IH) coaching; coaches (masters-level degrees in social work or psychology); telephone; individual	Usual care
2010, USA, Melkus [S12]	109	CBT	11	CBT+DSMT+CST; Nurse; face to face; group	Diabetes education; Nurse; face-to-face; group
2010, Belgium De Greef [S13]	41	CBT	5	Cognitive-behavioural pedometer-based group intervention; coaches (degree in PE, movement sciences or clinical psychology); face to face; group	Usual care
2010, USA, Hawkins [S14]	66	Counselling	12	Motivational interviewing video call; nurses, telephone, individual	Attention control telephone support (no MI); nurses; telephone; individual
2010, USA, Osborn [S15]	185	Counselling	1	Culturally tailored diabetes self-care intervention; bilingual medical assistant of Puerto Rican heritage; face to face; individual	Usual care

2011, Chile, Garcia-Huidobro [S16]	167	Counselling	4	Family intervention, Healthcare team, face-to-face, family	Usual care
2011, Ireland, Keogh [S17]	121	Counselling	3	Family-based intervention; Health psychologist; face to face; family	Usual care
2011, Belgium, De Greef [S18]	67	Counselling	3	1) Group behavioural intervention; clinical psychologist; face to face; group<br>2) individual consultation; GP, face to face, individual	Usual care
2011, Iran, Hamid [S19]	46	CBT	12	CBT, trained researcher, face to face, Group	Waiting list, see intervention description
2011, USA, Piette [S20]	291	CBT	12	Telephone delivered CBT; Nurses; telephone; individual	Enhanced usual care (usual care + copy of self-help book based on CBT for depression)
2011, Netherlands, Lamers [S21]	70	CBT	4	Minimal psychological intervention; Nurses; face to face; individual	Usual care
2011, USA, Welch [S22]	119	Counselling	4	1) MI +Computerized self-management: Diabetes educator; face to face; individual<br>2) MI alone; Diabetes educator; face to face; individual	1) Diabetes education alone; diabetes educator; face to face; individual<br>2) Computer self-management alone; computer; individual
2011, USA, Ell [S23]	229	CBT	Not reported	Sociocultural adapted collaborative care (relapse prevention): primary care physicians/graduate social workers/ diabetes depression clinical specialists (DDCS); face to face/telephone; individual	Enhanced usual care (usual care + prescribed antidepressant medication and provided counselling or refer to community mental health care.)
2012, UK, Farmer [S24]	211	Counselling	1	Consultation-based intervention, Clinical nurses, face-to-face, individual.	Usual care
2012, USA, Penckofer [S25]	74	CBT	8	Psychoeducation: Nurses; face to face, group	Usual care
2012, Germany, Hartmann [S26]	110	Counselling	8	Mindfulness-based intervention: psychologist and a resident in internal medicine; face to face; group	Usual care
2012, Taiwan, Chen [S27]	215	Counselling	Not reported	Motivational interviewing: Nurses; face to face; individual	Diabetes Education; nurse/diabetes educator; face to face; group
2013, Canada, Plotnikoff [S28]	287	Counselling	22	Telephone counselling (MI): five individuals with relevant degree qualifications related to PA promotion and/or counselling; telephone; individual	1) Diabetes education; Educational materials<br>2) Printed materials (relates to transtheoretical model)
2013, Netherlands, Welschen [S29]	154	CBT	3-6	CBT; diabetes nurse and dietician; face to face; individual	Usual care; dietician/diabetes nurse; face to face; individual
2013, USA, Mandel [S30]	131	CBT	4	Music therapy (relaxation and imagery); Music therapy clinician; face to face; group	1) Diabetes education; diabetes educator/dietician; face to face; group<br>2) music relaxation CD
2013, Netherlands,	521	Counselling	5-8	Motivational interviewing; Nurse; face-to-face; individual	Usual care

Jansink [S31]					
2014, Denmark, Juul [S32]	3946	Counselling	Variable	Nurse-led diabetes consultations, GP & nurses, face-to-face, individual.	Usual care
2014, UK, Steed [S33]	124	Counselling	5	Self-management intervention, Diabetes specialist nurse & dietician, face-to-face, group.	Usual care
2014, USA, Safren [S34]	87	CBT	9-12	CBT-AD: Therapist; face to face; individual	Enhanced usual care; nurse/dietician; face to face; individual
2014, Portugal, Gois [S35]	22	Interpersonal Psychotherapy (IPT)	12	Interpersonal Psychotherapy (IPT), Psychiatry, face-to-face, individual.	Medical care & sertraline
2014, China, Li [S36]	101	Counselling	4	Motivational interviewing; therapist; face to face; individual	Diabetes Education; face to face; individual
2014, UK, Griffin [S37]	478	Counselling	8	Intensive plus behavioural intervention: Life-style facilitators; face to face/telephone; individual	Enhanced usual care; GP; face to face; individual
2014, Australia, Eakin [S38]	277	Counselling	27	Telephone counselling (MI): trained researchers (degree nutrition or dietetics); telephone; individual	Usual care
2014, Netherlands, van Son [S39]	83	CBT	8	Mindfulness cognitive based therapy; psychologist; face to face; group	Usual care
2015, USA, Kim [S40]	209	Counselling	6	Self-management intervention, Nurses & community health workers, face-to-face, group.	Diabetes education, face-to-face, group.
2015, USA, Chlebowy [S41]	62	Counselling	4	Motivational interviewing: Nurses; face to face; individual	Usual care
2015, USA, Pladevall [S42]	1692	Counselling	6	Motivational interviewing and adherence information: Nurses and pharmacists; face to face/telephone; individual	1) Usual care<br>2) Adherence information; clinicians; face to face; individual
2015, Germany, Hermanns [S43]	60	CBT	5	DIAMOS: Psychologists, face to face; group	Diabetes Education; diabetes educators; face to face; group
2015, Croatia, Pibernik-Okanović [S44]	121	CBT	6	Psychoeducation: Psychologist; Face to face; Group	Diabetes Education; diabetologist; face to face; group
2015, Germany, Petrak [S45]	53	CBT	10	CBT, Clinical psychologists, face-to-face, group	Usual care and antidepressants
2016, Taiwan, Huang [S46]	61	CBT	12	MET+CBT: Psychotherapist/clinical nurse; face to face; Group	Usual care
2016, China, Browning [S47]	682	Counselling	9	Health coaching: Clinicians (doctors, nurses and psychologists; face-to-face/telephone; individual	Usual care
2016, Netherlands, Kasteleyn [S48]	161	Counselling	3	Motivational interviewing: Nurses; face to face; individual	Less intensive psychological intervention; nurse, telephone; individual
2016, Taiwan,	174	Counselling	4	Minimal Psychological Intervention: Psychology assistants; telephone;	Usual care

Chiu [S49]				individual	
2016, China, Fan[S50]	276	Counselling	3	Individualized diabetes education; Nurses and clinical psychologists; face to face; group	Diabetes education; nurses; face to face; group
2016, Denmark, Juul[S51]	127	Counselling	6	Health promotion intervention; Dietician, occupational therapist; face to face; group	Waiting list control
2016, Iran, Shayeghian [S52]	106	CBT	10	ACT; Clinical psychologists; face to face; group	Waiting list control
2016, USA, Wagner[S53]	107	Counselling	8	Stress management intervention; Community health worker; face to face; individual	Diabetes education; Community health worker; face to face; individual
2017, Turkey, Akturan[S54]	93	Counselling	3	BATHE interview technique; Physicians; face to face; individual	Usual care
2017, Italy, Balducci[S55]	300	Counselling	9	Counselling; Diabetologists and exercise specialists; face to face; individual	Usual care
2017, Malaysia, Chee[S56]	230	Counselling	1	1) Trans-cultural motivational interviewing; Dietician and physician; face to face; individual<br>2) Trans-cultural counselling; Dietician and physician; face to face; individual	Usual care
2017, USA, Egede[S57]	90	CBT	8	Behaviour activation treatment; therapists; face to face; individual	Behaviour activation treatment; therapists; face to face; video teleconferencing
2017, Australia, Furler[S58]	266	Counselling	Variable	The Stepping Up model of care intervention; nurses; face to face; individual	Usual care
2017, Germany, Hermanns[S59]	160	Counselling	6	Self-management-oriented education programme; Diabetes educators; face to face; group	Diabetes education; diabetes educators; face to face; group
2017, Spain, Munoz-Florez[S60]	26	Counselling	Variable	Motivational interviewing; Psychologist; face to face; individual	Educational materials and usual care
2017, Australia, Rees[S61]	40	CBT	8	Problem-solving therapy; Research assistant trained in PST supervised by clinical psychologist; telephone and face to face; individual	Usual care
2017, USA, Carrasquillo[S62]	300	Counselling	Variable	Community Health Worker Intervention; Community health workers; telephone & face to face; individual	Enhanced usual care (usual care +education materials)
2017, Brazil, Gomes[S63]	222	Counselling	4	Family social support; families; telephone; family	Education; telephone; individual
2017, China Jiang[S64]	52	Counselling	Not reported	Problem-solving treatment, face-to-face, group	Usual care plus paroxetine
2018, Malaysia,	124	Counselling	4	VEMOFIT (emotion focused education programme); Nurse and	Attention control; Nurse and physician; face to face;

Chew[S65]				physician; face to face; group	group
2018, USA, Chwastiak[S66]	29	Counselling	12	Collaborative care; Nurse case manager, psychiatrist, advanced practice nurse; face to face; individual	Usual care
2018, Germany, Dobler[S67]	199	Counselling	12	Telephone support group; Counsellors; face to face; individual	Usual care
2018, UK, Ismail[S68]	334	Counselling	12	D6 (MI+CBT); nurses; face to face; individual	Usual care
2018, Iran, Momtzi[S69]	30	Counselling	4	Motivational interviewing; Psychiatrist; face to face; group	Waiting list control
2018, UK, Wroe[S70]	115	CBT	6	Wellbeing Group; IAPT practitioners; face to face; group	Usual care
			**Studies included in systematic review only**		
2004, UK, Clark [S71]	100	Counselling	1	Self-management intervention: Interventionist (trained in MI); face to face; individual	Usual care
2004, Norway, Karlsen [S72]	63	CBT	9	Group-based counselling; nurse; face to face; group	Waiting list
2006, USA, Hokanson [S73]	114	Counselling	4-7	Smoking cessation motivational interviewing, research staff, telephone, individual	Usual care
2010, The Netherlands, Heinrich [S74]	537	Counselling	8	Motivational interviewing; nurses; face to face; individual	Usual care
2010, Iran, Pourisharif [S75]	41	Counselling	4	1) Motivational interviewing; face to face; group<br>2) CBT; face to face; group	Usual care
2011, Italy, Castelnuovo [S76]	34	CBT	Variable	TECNOB (TEChnology for OBesity): Clinical psychologist; Face to face/telephone/ online and text messaging; individual/ group	Usual care
2012, USA, Waker [S77]	154	Counselling	2	Motivational interviewing: researcher; face to face; individual	Usual care
2013, USA, Gabbay [S78]	545	Counselling	8	Motivational interviewing: Nurses; face to face; individual	Usual care

2015, USA, Inouye [S79]	207	CBT	6	CBT: Research assistants; face to face; Group	Diabetes education; research assistants; face to face; group
2016, USA, Fitzpatrick [S80]	182	Counselling	9	1) DECIDE Group, graduate assistant, face-to face, group<br>2) DECIDE individual, graduate assistant, face-to face, individual	1) Enhance usual care (usual care + education materials), face-to-face/mail, individual<br>2) DECIDE self-study; mail; individual
2016, Netherlands, Rondags[S81]	14	Counselling	3	Blood glucose awareness training; diabetes professionals; face-to-face; group	Usual care
2017, Canada, Cummings[S82]	129	CBT	16	Lifestyle coaching; Peers; face to face; telephone	Usual care
2017, USA, Egede[S83]	255	Counselling	12	Telephone-Delivered Behavioural Skills Intervention (knowledge, skills, or knowledge & skills); health educators; telephone; individual	Usual care
2017, New Zealand. Friis[S84]	17	Counselling	8	Mindful self-compassion (MSC) intervention; health psychologists; face-to-face; group	Waiting list
2017, Finland, Tovote[S85]	56	CBT	8	Mindfulness-Based Cognitive Therapy; CBT therapists; face to face; individual	Waiting list
2017, New Zealand, Whitehead[S86]	97	CBT	1	Education + ACT; clinical psychologist & nurses; face to face; group	1) Diabetes education; nurses; face to face; group<br>2) Usual care
2018, Netherlands, Berk[S87]	158	CBT	14	Group cognitive behavioural therapy; trained psychologist/psychotherapist, with experience in diabetes care; face to face; group	Usual care
2018, Iran, Kian[S88]	59	Counselling	8	Mindfulness-Based Stress Reduction; Mindfulness instructor; face to face; group	Usual care
2018, USA, Pyatak[S89]	19	Counselling	Variable	Occupational therapy; occupational therapists; face to face; individual	Attention control follow-up phone calls
2014, USA, Lin [S90]	Not reported	Counselling	Variable	Collaborative care; Primary care physician & nurse & psychiatrist & psychologist; face to face; individual	Usual care
2011, Denmark, Minet [S91]	349	Counselling	5	Motivational interviewing; HCPs (nurse, dietician, physiotherapist or psychologist); face to face; individual	Usual care
2015, USA, Safford [S92]	Not reported	Counselling	Variable	Motivational interviewing; peers, telephone; individual	Diabetes education; face to face; individual
2015, Netherlands, Schroevers [S93]	24	CBT	8	Mindfulness-based cognitive therapy (MBCT); clinical psychologist; face to face; individual	Waiting list
2011, USA, Weinger [S94]	222	CBT	5	Structured behavioural group; diabetes educators; face to face, group	1) Group attention control; diabetes educators; face to face, group<br>2) Individual control; diabetes educators; face to face; individual

Table S3 – Case definition of included meta-analysed studies

Year, Country, reference	Mean age (SD or range), years intervention group	Mean age (SD or range), years control group	Mean (SD or range) duration of diabetes, years intervention group	Mean (SD or range) duration of diabetes, years control group	Mean (SD) baseline HbA1c intervention group	Mean (SD) baseline HbA1c control group	Age inclusion criteria (years)	Diabetes duration inclusion criteria (months)	HbA1c inclusion criteria
2004, USA, Whittemore [S1]	All: 57·6 (10·9)	All: 57·6 (10·9)	All: 2·7 (3·0)	All: 2·7 (3·0)	7.7% (1.00)	7.6% (1.00)	30-70	None	7% or more
2004, USA, Williams [S2]	70·1 (6·9)	70·3 (7·1)	NR	NR	7.26% (1.32)	7.30% (1.43)	≥60	None	None
2006, Germany, Siebolds [S3]	58·7 (7·6)	60·5 (6·6)	65·5 (57·2)	62·6 (47·3)	8.47% (0.86)	8.35% (0.75)	≥18	None	None
2006, Thailand, Keeratiyutawong [S4]	NR	NR	NR	NR	8.93% (2.4)	7.89% (1.8)	21-60	<120	None
2007, USA, Gregg [S5]	49·8	49.8	5·3	6·6	8.17% (1.86)	8.21% (1.91)	≥18	None	None
2007, USA, West [S6]	54.0 (10.0)	52.0 (10.0)	5·8 (6·5)	4·9 (5.0)	7.54% (1.4)	7.62% (1.4)	≥18	None	None
2009, UK, Dale [S7]	NR	NR	NR	NR	1) 8.9% (1.5)<br>2) 8.4% (1.1)	8.7% (1.3)	≥18	None	>7.4%
2009, Iran, Davazdah [S8]	NR	NR	NR	NR	7.04% (1.39)	7.51% (1.53)	≥18	None	None
2009, USA, Sacco [S9]	All: 52(8·6)	All: 52(8·6)	All: 9·5 (7·2)	All: 9·5 (7·2)	8.4% (1.37)	8.5% (2.01)	18-65	None	None
2010, Australia, Evans [S10]	All: 57·1(22-84)	All: 57·1(22-84)	All: 14·3(1-45)	All: 14·3(1-45)	8.33% (1.44)	7.41 (1.64)	≥18	None	None
2010, USA, Wolever [S11]	53·1 (8·29)	52·8(7·64)	11·8 (8·5)	10·6 (6·43)	7.7% (1.94)	8.2% (1.89)	≥18	≥12	None
2010, USA, Melkus [S12]	47.0 (9.0)	45.0 (10.0)	NR	NR	8.0% (2.1)	8.3% (2.2)	21-65	None	None
2010, Belgium De Greef [S13]	NR	NR	NR	NR	7.5% (1.1)	8.0% (1.3)	35-75	≥6	None

2010, USA, Hawkins [S14]	64	65·8 (10·4)	NR	NR	9.0% (2.3)	8.9% (3.1)	≥60	None	7% or more
2010, USA, Osborn [S15]	56·9 (11·3)	58·4 (10·1)	13·2 (12)	12·3 (9·4)	7.8% (1.4)	7.3% (1.6)	≥18	≥12	None
2011, Chile, Garcia-Huidobro [S16]	53·4 (8·1)	53·5 (9·8)	NR	NR	10.3% (2.0)	9.5% (2.2)	18-70	None	7% or more
2011, Ireland, Keogh [S17]	59·96 (11·67)	57·29 (11·34)	9·17(7·1)	9·65 (6·45)	Median: 9.06 (0.96)	Median: 9.29 (1.13)	>18	>12	8% or more
2011, Belgium, De Greef [S18]	I1: 70 (6·3)<br>I2: 66·6 (9·5)	66 (11·1)	NR	NR	I1: 7.23% (0.71)<br>I2: 7.12% (1.35)	7.0 (0.87)	<80	≥6	<12%
2011, Iran, Hamid [S19]	NR	NR	NR	NR	9.03% (3.1)	9.1% (3.6)	≥18	None	None
2011, USA, Piette [S20]	55·1 (9·4)	56 (10·9)	NR	NR	7.5% (1.7)	7.7% (1.7)	≥21	None	None
2011, Netherlands, Lamers [S21]	70·7 (6·6)	69·7 (6·6)	8·2 (8·8)	9·8 (9·1)	7.5% (1.1)	7.2% (1.4)	≥60	None	None
2011, USA, Welch [S22]	I1: 56·1 (10·4)<br>I2: 54·9 (9·3)	C1: 57·2 (10·9)<br>C2: 54·4 (10·3)	I1: 9·8 (8)<br>I2: 9 (7·3)	C1: 7 (6·5)<br>C2: 7·1 (5·8)	I1: 9.1 (1.5)<br>I2: 8.8 (1.0)	C1: 8.8 (1.3)<br>C2: 8.9 (1.2)	30-70	None	7.5% or more
2011, USA, Ell [S23]	All: 54 (8·7)	All: 54 (8·7)	NR	NR	9.03% (2.23)	9.13% (2.21)	≥18	None	None
2012, UK, Farmer [S24]	62·5 (11.0)	64·1 (10·3)	6·7 (4·8)	6·9 (5·3)	8.37% (1.25)	8.28% (1.22)	≥18	≥3	7.5% or more
2012, USA, Penckofer [S25]	54·8 (8·8)	54 (8·4)	10·5 (8·2)	10 (6·5)	7.8% (1.8)	7.9% (2.0)	≥18	≥6	None
2012, Germany, Hartmann [S26]	58·7 (7·4)	59·3 (7·8)	11.0 (7·5)	12·2 (7·6)	7.26% (1.08)	7.27% (1.06)	30-70	<36	None
2012, Taiwan, Chen [S27]	59·19 (10·24)	58·67 (10·23)	7·98 (7·57)	7·91 (6·95)	8.92% (2.17)	8.52% (1.82)	>18	>3	None
2013, Canada, Plotnikoff [S28]	62·3 (11·1)	C1: 61.0 (11·7)	8·8 (7.0)	C1: 11·7 (9·9)<br>C2: 10·7 (9·9)	7.08% (1.3)	C1: 7.06% (1.9)	≥18	None	None

		C2: 61·4 (12·6)				C2: 7.24% (0.12)			
2013, Netherlands, Welschen [S29]	60·5 (9·4)	61·2 (8·8)	7·6 (5)	7·8 (6·1)	6.8% (1.0)	6.7% (1.0)	18-75	None	7% or more
2013, USA, Mandel [S30]	58 (11·29)	C1: 57·1 (9·67)<br>C2: 58·9 (10·76)	3·22 (5·94)	C1: 2·32 (6·1)<br>C2: 3·78 (7·06)	7.7% (1.81)	C1: 7.4% (1.56)<br>C2: 7.6% (1.48)	30-85	None	None
2013, Netherlands, Jansink [S31]	64·1 (8·9)	63·9 (9·8)	7·5 (6·0)	7·8 (5·8)	7.8% (0.9)	7.7% (0.7)	<80	None	7% or more
2014, Denmark, Juul [S32]	60·2 (8·2)	60·7 (8·6)	8 (4-14)	8 (4-15)	7.1% (1.3)	7.1% (1.3)	40-74	None	None
2014, UK, Steed [S33]	59·2 (8·8)	60·3 (8·6)	10·7 (7·5)	10·9 (7·9)	8.2% (1.3)	8.6% (1.8)	< 75	None	None
2014, USA, Safren [S34]	55·44 (8·72)	58·31 (7·41)	NR	NR	8.81% (1.78)	8.74% (1.41)	18-70	None	7% or more
2014, Portugal, Gois [S35]	56·82 (4·25)	53·81 (7·04)	13·12 (4·85)	11·63 (6·68)	9.36% (2.38)	8.76% (1.94)	18-65	>6	None
2014, China, Li [S36]	58·5 (5.0)	59·2 (5·2)	1·3 (0·5)	1·2 (0·4)	10.1% (2.7)	9.7% (3.5)	40-70	12-24	9% or more
2014, UK, Griffin [S37]	59·5 (7·5)	59·8 (7·5)	NR	NR	7.23% (1.62)	7.01% (1.23)	40-69	< 36	None
2014, Australia, Eakin [S38]	57·7 (8·1)	58·3 (9·0)	NR	NR	Median: 7.6% (6.3, 8.5)	Median: 7.0% (6.4, 7.9)	20-75	None	None
2014, Netherlands, van Son [S39]	56.0 (13.0)	57.0 (13.0)	NR	NR	7.5% (1.2)	7.6% (1.2)	18-80	None	None
2015, USA, Kim [S40]	59·1 (8·4)	58·3 (8·5)	In months: 105·3 (87·6)	In months: 99·3 (84·8)	8.9% (2.05)	8.8% (3.06)	≥35	None	7% or more
2015, USA, Chlebowy [S41]	55·8 (2·1)	53 (2·25)	NR	NR	7.8% (0.16)	8.1% (0.18)	≥18	None	None

2015, USA, Pladevall [S42]	64·5 (10·5)	C1: 64·9 (11·5)<br>C2: 63·3 (10·9)	NR	NR	8.0% (1.3)	C1: 8.2% (1.4)<br>C2: 8.0% (1.4)	≥18	None	7% or more
2015, Germany, Hermanns [S43]	34·2 (14·9)	43·4 (13·8)	14·2 (10·3)	14·2 (10·7)	8.9% (1.8)	8.9% (1.8)	18-70	None	None
2015, Croatia, Pibernik-Okanović [S44]	57·7 (6·2)	58·2 (5·6)	11·4 (9·1)	10·5 (6·9)	7.4% (1.2)	7.2% (1.1)	18-65	≥12	None
2015, Germany, Petrak [S45]	49 (10·6)	47·9 (12·8)	15·7 (10·4)	15·0 (10·6)	9.30% (1.49)	9.20% (1.44)	21-69	None	7.5% or more
2016, Taiwan, Huang [S46]	55·06 (10·44)	57·83 (10·38)	In months:<br>44·32 (21·59)	In months:<br>45·7 (18·06)	7.68% (1.44)	7.84% (1.95)	≥20	None	None
2016, China, Browning [S47]	63·7 (7·6)	64.0 (9.0)	10.0 (6·5)	9·6 (6·6)	10.60% (2.09)	10.29% (1.71)	≥50	None	None
2016, Netherlands, Kasteleyn [S48]	66.0 (9·3)	65·6 (9·4)	7.0 (2·8-16)	8·5 (5-15)	7.2% (3.5)	6.8% (3.1)	>35	>12	None
2016, Taiwan, Chiu [S49]	64·78 (0·3)	64·59 (0·4)	10.0 (0·6)	10·58 (0·2)	7.6% (1.5)	7.7% (1.3)	≥50	None	None
2016, China, Fan[S50]	62·94 (10·72)	64·89 (10·14)	11·4 (4·8)	11·6 (5·0)	9.61% (1.92)	9.80% (1.98)	None	None	None
2016, Denmark, Juul[S51]	Median:<br>58 (50, 63)	Median:<br>60 (51, 64)	NR	NR	40.7 mmol/mol (3.5)	40.6 mmol/mol (3.9)	<70	None	6-6.4%
2016, Iran, Shayeghian[S52]	55·18 (8·26)	55·70 (8·98)	4·9 (1·40)	4·54 (1·54)	7.46% (1.66)	7.61% (1.38)	40-60	12-120	None
2016, USA, Wagner[S53]	60.0 (11·2)	60·8 (12·1)	NR	NR	8.5% (1.4)	8.6% (1.9)	≥18	≥6	7% or more
2017, Turkey, Akturan[S54]	57·51 (7.0)	56·33 (7·56)	NR	NR	7.48% (1.49)	7.39% (1.28)	18-80	≥6	None
2017, Italy, Balducci[S55]	NR	NR	NR	NR	7.43% (1.60)	7.32% (1.37)	40-80	≥12	None

2017, Malaysia, Chee[S56]	NR	NR	NR	NR	I1: 7.7% (1.1)<br>I2: 7.7% (1.4)	7.9% (1.3)	30-65	None	7-11%
2017, USA, Egede[S57]	62·7 (3·4)	63·5 (4·9)	NR	NR	7.35%	6.90%	≥58	None	None
2017, Australia, Furler[S58]	61·7 (9·7)	62·0 (10·6)	NR	NR	8.7% (8.1-9.7)	8.5% (8-9.6)	<80	None	7.5% or more
2017, Germany, Hermanns[S59]	NR	NR	NR	NR	8.0% (1.3)	7.9% (1.2)	18-75	None	None
2017, Spain, Munoz-Florez[S60]	66.0 (9·45)	63.0 (12·82)	NR	NR	156.3 mg/DL (47.79)	134 mg/DL (46.6)	≥20	None	None
2017, Australia, Rees[S61]	60·1 (7.0)	58·6 (8·8)	17·5 (10)	23.0 (15.0)	8.2% (1.57)	8.1% (1.2)	None	None	None
2017, USA, Carrasquillo [S62]	55·3 (7·1)	55·2 (6·1)	11·7 (8·2)	11·2 (8·4)	9.3% (2.1)	9.3% (1.9)	18-65	≥6	8% or more
2017, Brazil, Gomes[S63]	NR	NR	NR	NR	9.47% (2.01)	9.40% (2.00)	≥40	None	None
2017, China Jiang[S64]	56·3 (5·3)	57·1 (5·5)	1·24 (0·38)	1·27 (0·36)	7.7% (0.9)	8.3% (1.1)	18-70	None	None
2018, Malaysia, Chew[S65]	55·6 (10·8)	55·8 (8·8)	NR	NR	9.9% (1.8)	9.5% (2.1)	≥18	≥24	8% or more
2018, USA, Chwastiak[S66]	NR	NR	NR	NR	9.4% (2.2)	8.3% (1.9)	18-64	≥6	8% or more
2018, Germany, Dobler[S67]	51·6 (5·7)	52·2 (5·4)	8·7 (6·6)	9·6 (5·9)	7.8% (1.7)	7.6% (1.4)	18-70	None	None
2018, UK, Ismail[S68]	59 (11·1)	58·9 (11·4)	10.0 (7-13)	9.0 (5-12)	81.0 mmol/mol (17.1)	80.1 mmol/mol (19.1)	18-79	≥24	8% or more
2018, Iran, Momtzi[S69]	NR	NR	NR	NR	8.23% (1.10)	7.98% (0.80)	30-60	None	7% or more
2018, UK, Wroe[S70]	63·48 (11·04)	63·63 (10·71)	NR	NR	67.12 mmol/mol (21.02)	61.86 mmol/mol (14.29)	None	None	None

Table S4 – Primary outcome of included meta-analysed studies

Reference	Primary outcome category	Primary outcome description
Whittemore et al. 2004	Self-management	Diet self-management
Williams et al. 2004	HbA1c	HbA1c
Keeratiyutawong et al. 2006	HbA1c	HbA1c
Gregg et al. 2007	HbA1c	HbA1c
West et al. 2007	Biomedical	Weight
Dale et al. 2009	Psychological	Self-efficacy
Davazdah et al. 2009	HbA1c	HbA1c
Sacco et al. 2009	Self-management	Medication adherence
De Greef et al. 2010	Self-management	Physical activity
Osborn et al. 2010	Self-management	Diet adherence
Evans et al. 2010	Psychological	Depression
Hawkins et al. 2010	HbA1c	HbA1c
Wolever et al. 2010	Self-management	Medication adherence
Melkus et al. 2010	HbA1c	HbA1c
Keogh et al. 2011	HbA1c	HbA1c
Welch et al. 2011	HbA1c	HbA1c
Piette et al. 2011	HbA1c	HbA1c
Garcia-Huidobro et al. 2011	HbA1c	HbA1c
Lamers et al. 2011	Psychological	Depressive symptoms
Ell et al. 2011	Psychological	Depressive symptom treatment
De Greef et al. 2011	Self-management	Physical activity
Hamid et al. 2011	HbA1c	HbA1c
Farmer et al. 2012	Self-management	Medication adherence
Hartnamnn et al. 2012	Psychological	Depressive symptoms
Penckofer et al. 2012	Psychological	Depression
Chen et al. 2012	HbA1c	HbA1c
Welschen et al. 2013	Biomedical	Coronary heart disease risk
Jansink et al 2013	HbA1c	HbA1c
Mandel et al. 2013	HbA1c	HbA1c
Plotnikoff et al. 2013	HbA1c	HbA1c
Gois et al. 2014	Psychological	Depressive symptoms
Steed et al. 2014	Psychological	Self-efficacy
Siebolds et al. 2006	HbA1c	Hba1c
Juul et al. 2014	HbA1c	HbA1c
Eakin et al. 2014	Biomedical	Weight loss
Safren et al. 2014	Self-management	Medication adherence

Van Son et al. 2014	Psychological	Stress
Griffin et al. 2014	Self-management	Physical activity
Li et al. 2014	HbA1c	HbA1c
Chlebowy et al. 2015	Self-management	Medication adherence
Petrak et al. 2015	HbA1c	HbA1c
Hermanns et al. 2015	Psychological	Depressive symptoms
Pladevall et al. 2015	HbA1c	HbA1c
Pibernik-Okanović et al. 2015	Psychological	Depressive symptoms
Kim et al. 2015	HbA1c	HbA1c
Browning et al. 2016	HbA1c	HbA1c
Fan et al. 2016	Biomedical	BMI
Wagner et al. 2016	Psychological	Depressive symptoms
Huang et al. 2016	HbA1c	HbA1c
Shayeghian et al. 2016	HbA1c	Hba1c
Kasteleyn et al. 2016	Psychological	Diabetes distress
Chiu et al 2016	Psychological	Diabetes distress
Juul et al. 2016	Biomedical	Weight loss
Jiang et al. 2017	HbA1c	HbA1c
Chee et al. 2017	HbA1c	HbA1c
Balducci et al. 2017	Self-management	Physical Activity
Hermanns et al. 2017	HbA1c	HbA1c
Rees et al. 2017	Psychological	Diabetes distress
Akturan et al. 2017	Psychological	Diabetes Empowerment
Furler et al. 2017	HbA1c	HbA1c
Carrasquillo et al. 2017	HbA1c	HbA1c
Gomes et al. 2017	HbA1c	HbA1c
Egede et al. 2017	Psychological	Depressive symptoms
Munoz-Florez et al. 2017	Self-management	Physical activity
Dobler et al. 2018	Self-management	Physical activity
Momtazi et al. 2018	HbA1c	HbA1c
Ismail et al. 2018	HbA1c	HbA1c
Wroe et al. 2018	Psychological	Depressive symptoms
Chew et al. 2018	Psychological	Diabetes Distress
Chwastiak et al. 2018	HbA1c	HbA1c

Winkley K, *et al. BMJ Open Diab Res Care* 2020; 8:e001150. doi: 10.1136/bmjdrc-2019-001150

Table S5- Frequency of studies with co-morbid depressive symptoms inclusion criteria and HbA1c as a primary outcome

	**Primary outcome= HbA1c**		
**Inclusion criteria=depressive symptoms**	**Yes**	**No**	**Total**
**Yes**	6	10	16
**No**	27	27	54
**Total**	33	37	70

Winkley K, *et al. BMJ Open Diab Res Care* 2020; 8:e001150. doi: 10.1136/bmjdrc-2019-001150

Table S6 – Additional inclusion/exclusion criteria information

Reference	Inclusion/exclusion based on mental health or cognitive impairment	Other inclusion criteria	Other exclusion criteria
Whittemore et al. 2004		Female, able to exercise, no advanced diabetes complications, fluent in English, previously participated in diabetes education.	
Williams et al. 2004	Included if systematic depression screening with a 2-item depression screener adapted from the Primary Care Evaluation of Mental Disorders.		
Keeratiyutawong et al. 2006		Only oral diabetes meds, fasting blood glucose >130mg for 2 times or more, read Thai.	Excluded if on insulin therapy, presence of other serious illness or complications relating to diabetes.
Gregg et al. 2007		English-speaking, receiving medical care at low-income community health centre, and referred to diabetes education.	
West et al. 2007		Treated with OADs not insulin, BMI 27-50, able to walk for exercise.	Excluded if pregnant, recent significant weight loss (>10 lbs), or a severe debilitating disease that might interfere with study participation.
Dale et al. 2009	Exclude if severe accompanying disorders (e.g. mentally ill).	Not on insulin, Speak English; no severe accompanying disorders (e.g. mentally ill, severe learning difficulties, severe hearing difficulties).	
Davazdah et al. 2009	Included if presence of depressive symptoms according to DASS scale.		
Sacco et al. 2009	Excluded if evidenced major mental disorder (e.g. schizophrenia) that would potentially interfere with implementation of intervention.	Able to speak and read English, at least one of the following cardiovascular risk factors (low-density lipoprotein =100 mg/dl; high-density lipoprotein=40 mg/dl for men or =45 mg/dl for women; triglycerides =150; cholesterol/high-density lipoprotein ratio =5/1).	
De Greef et al. 2010		No physical activity limitations.	

Osborn et al. 2010		Puerto Rican ethnicity.	
Evans et al. 2010	Borderline personality disorder		
Hawkins et al. 2010			Excluded if unable to pass the Short Portable Mental Status Questionnaire (SPMSQ)
Wolever et al. 2010	Excluded if presence of dementia, Alzheimer, schizophrenia, cognitive impairment.	Have taken OADs for at least 1 year, not on insulin.	
Melkus et al. 2010	Excluded if presence of serious psychiatric disorder.	Black women, not on insulin, BMI<37, not pregnant.	Excluded if diagnosed with a serious medical condition (cancer, AIDS), diabetes related complications.
Keogh et al. 2011			
Welch et al. 2011	Excluded if presence of severe psychiatric disorders or mental retardation, or visual, literacy, or comprehension barriers that would prevent completion of study questionnaires.	Able to speak or write in English.	Excluded if diagnosed with major diabetes complications, or pregnant.
Piette et al. 2011	Included if presence of depressive symptoms according to PHQ (score of 11 or more). Excluded if diagnosed with bipolar disorder or schizophrenia.	Prescribed antihyperglycemic medication.	Excluded if not using antihyperglycemic medication, had been diagnosed with or were in active treatment for another serious illness such as severe heart failure, severe chronic obstructive pulmonary disease, or end-stage renal disease.
Garcia-Huidobro et al. 2011	Excluded if diagnosed with cognitive disorder which limits participation.	Live in household with a significant family member >15yrs.	Excluded if hospitalised during 3m prior HbA1c measurement.
Lamers et al. 2011	Included if presence of depressive symptoms according to MINI (Mild to moderate major depression). Excluded if treatment with antidepressants for depression or present , major psychiatric problems (bipolar depression, schizophrenia, alcohol or substance abuse), current psychosocial/psychiatric treatment, serious cognitive problems.		Excluded if on waiting list for nursing home, bedridden, loss of spouse in last 3 months and not being fluent in Dutch.
Ell et al. 2011	Included if presence of depressive symptoms according to PhQ-9 (one of the two cardinal		

	depression symptoms more than half the days to nearly every day over the last 2 weeks and scored ≥10 on the) PhQ-9. Excluded if present acute suicidal ideation, alcohol abuse, self-reported recent lithium/antipsychotic medication use.		
De Greef et al. 2011		BMI 25-35; pharmaceutically treated for type 2 diabetes; no physical limitations; Speak Dutch.	
Hamid et al. 2011	Included if presence of depressive symptoms according to DASS scale.		
Farmer et al. 2012		Taking OADs (not excluded if taking insulin).	
Hartnamnn et al. 2012	Excluded if presence of psychiatric disorders.		Excluded if presence of albuminuria, non-diabetic kidney disease, alcohol or drug abuse, malignant tumours, heart failure, acute coronary syndrome.
Penckofer et al. 2012	Included if presence of depressive symptoms according to CES-D score (16 more more). Excluded if a history of bipolar depression, or any other psychotic disorder.		Excluded if current alcohol or substance abuse disorders, a diabetes knowledge score <70 % on the Brief Diabetes Knowledge Test (since the program emphasis was not diabetes education); and severe complications of diabetes (blindness, renal failure, or amputation).
Chen et al. 2012	Excluded if presence of psychiatric illness.	Speak Chinese.	Excluded if too ill due to terminal illness or haemodialysis.
Welschen et al. 2013		Able to understand Dutch language, high risk of developing CVD and diabetes complications (HbA1c = 52 mmol/mol (7.0 %) and/or body-mass index = 27.0 kg/m2 and/or smoking).	
Jansink et al 2013		BMI>25.	Exclusion if presence of complex comorbidity and receiving treatment in hospital.
Mandel et al. 2013			Excluded if diagnosed with gestational diabetes, dementia, severe hearing loss.
Plotnikoff et al.		Access to telephone, no English language	

2013		barrier	
Gois et al. 2014	Included if presence of depressive symptoms according to HADs score (7 or more on depression sub-scale), MADRS (score of 17 or more points), and major depression diagnosis using MINI and DSM-IV. Excluded if history of psychotic disorder or have regular psychoactive medications, active suicidal ideation.		Excluded if presence of severe complications that interfere with self-care activities, other chronic physical disease, alcohol or drug abuse.
Steed et al. 2014		Presence of microalbuminuria as indicated by two or more urinary albumin to creatinine ratios >3.0 mg/mmol or a urinary albumin excretion >30 mg/24h, fluency in spoken English	
Siebolds et al. 2006			
Juul et al. 2014			
Eakin et al. 2014		Physically inactive, BMI 25 or more, not using weight loss medications, without previous or planned bariatric surgery	
Safren et al. 2014	Included if presence of depressive symptoms according to DSM-IV. Excluded if severely depressed (requiring intensive treatment such as hospitalisation). Excluded if untreated major mental illness (e.g., untreated psychosis), bipolar disorder, eating disorder, mental retardation, dementia, or active suicidality or were undergoing current CBT for depression were excluded.		Excluded if unable or unwilling to provide informed consent.
Van Son et al. 2014	Included if Poor emotional well-being (<13 score on WHO-5). Excluded if a recent history of severe psychopathology (i.e., psychosis, risk of suicide attempts), or were already in an (extensive) psychological treatment which started within a period of 6 week before the		Excluded if alcohol/drugs abuse; have a severe physical co-morbidity (i.e., severe forms of cancer or heart failure); when they have insufficient reading and comprehension skills of the Dutch language.

	start of the training.		
Griffin et al. 2014	Excluded if had a psychotic illness.		Excluded if had an illness with a likely prognosis of <1 year; women pregnant.
Li et al. 2014		Education level of at least 6 years.	Excluded if disturbance of consciousness, cognitive disorders or defects in language communication; presence of a severe acute disease or chronic disease (e.g. severe heart failure, lung function failure, tumors).
Chlebowy et al. 2015	Excluded if receiving treatment form a mental health provider.	African American ethnicity; treated by OADs or insulin; English speaking; able to engage in moderate physical activity.	
Petrak et al. 2015	Included if presence of major depression according to DSM-IV. Excluded if suicidal ideations, psychotic symptoms, bipolar disorder, substance abuse or dependence in the past 6 months, psychotherapy in the preceding 3 months, current use of mood stabilizers neuroleptics, antidepressants, or benzodiazepines.	Insulin treated.	Excluded if liver enzyme elevations to exclude severe liver dysfunction.
Hermanns et al. 2015	Included if presence of depressive symptoms CES-D (score of 16 or more). Excluded if presence of major depression, current schizophrenia/psychotic disorder, eating disorder, bipolar disorder, addictive disorder, or personality disorder; current use of antidepressant medication or ongoing psychotherapy.	Sufficient German language skills.	Excluded if bedridden; and under guardianship.
Pladevall et al. 2015		1 or more HbA1c 7% or more ≥1 LDL-C measurement with the last value ≥100 mg/dL, and ≥1 prescription for both an oral diabetes medication and a lipid-lowering medication.	
Pibernik-Okanović et al. 2015	Included if presence of depressive symptoms according to PhQ-2 (1 depressive symptom over past month & need for professional help). Excluded if presence of major depression, or		Excluded if diagnosed with advanced diabetes complications, medical contraindications for physical exercise.

Winkley K, et al. BMJ Open Diab Res Care 2020; 8:e001150. doi: 10.1136/bmjdrc-2019-001150

	dysthymia, as determined by phone-administered Structured Clinical Interview of DSM-IV, current psychiatric treatment.		
Kim et al. 2015		Korean American immigrant.	
Browning et al. 2016		Lived in Fengtai district, had health record at participating health services.	
Fan et al. 2016	Excluded if any known psychological or psychiatric disorders, such as major depression or generalize anxiety disorders.		Excluded if severe co-morbidities such as renal failure, hepatic dysfunction, cancer or stroke; uncontrolled complications from diabetes.
Wagner et al. 2016	Excluded for bipolar disorder or thought disorder; or suicide attempt or psychiatric hospitalization in the past 2 years.	Latino or Hispanic, Spanish-speaking.	Excluded for medical instability or intensive medical treatment.
Huang et al. 2016	Included if presence of depressive symptoms according to CES-D (score of 16 or more).	Exclusion= alcohol or drug abuse or dependence, clinically diagnosed neurological illness such as dementia, medical illness, and physical impairments severely influencing the individual's cognitive dysfunction.	
Shayeghian et al. 2016		No change in diabetes medication for 3 months before entering study.	Excluded if hospitalised or diagnosed with diabetes complications.
Kasteleyn et al. 2016		Speak Dutch, no serious illness to prevent participation.	
Chiu et al 2016	Included if occasional distress or minor depressive symptoms. Excluded if on anti-depressant medication, receiving ongoing psychological/psychiatric treatment, diagnosed with psychosis, severe cognitive problem.		Excluded if hearing impairment, lost partner within the past three months.
Juul et al. 2016		Impaired fasting glucose.	
Jiang et al. 2017	Excluded if presence of psychological disorders.	BMI>30.	Excluded if diagnosed with diabetes complications, severe visceral organ disease.
Chee et al. 2017		BMI>23; not treated with insulin, diabetes treatment not changed in last 3 months; nor pregnant; no history of serious diabetes complications	

Balducci et al. 2017		BMI 27-40; physical inactivity; sedentary lifestyle for at least 6m; able to walk 1.6km without assistance	
Hermanns et al. 2017	Excluded if presence of psychiatric disorders, dementia, severe cognitive impairment.	Treated with OADs or 2 years or more, non-intensified insulin treatment, BMI 20-40, read and understand German.	Excluded if severe disease complications or gestational diabetes.
Rees et al. 2017	Included if presence of diabetes distress according to the DDS (score of 2 or more. Excluded if insufficient cognitive ability to engage in study.	Diagnosis of diabetic retinopathy.	Excluded if insufficient English language.
Akturan et al. 2017	Excluded if diagnosed or treated for depression.		
Furler et al. 2017	Excluded if presence of severe mental illness.	Max OAD treatment (2 OADs at max dose).	Excluded if complex debilitating medical condition, such as end stage cancer, or unstable cardiovascular disease.
Carrasquillo et al. 2017		Latino.	
Gomes et al. 2017		Lack of advanced complications, other serious diseases that can prevent participation.	
Egede et al. 2017	Included if presence of depressive symptoms according to DSM-IV criteria for major depressive disorder. Excluded if diagnosis with active psychosis, dementia, suicidal ideation with clear intent, or substance dependence.		
Munoz-Florez et al. 2017		Ability to exercise (low to moderate activity every week).	
Dobler et al. 2018		Speak German; no acute substance-related disorder.	
Momtazi et al. 2018		At least high school diploma, taking oral diabetes medications.	
Ismail et al. 2018	Excluded if presence of severe mental disorders (PhQ-9 >20 if psychotic depression or active suicidal ideation) or receiving psychological treatment elsewhere.		Excluded if diagnosed with a terminal illness and end-stage diabetes complications, BMI>40, non-English.

Winkley K, *et al. BMJ Open Diab Res Care* 2020; 8:e001150. doi: 10.1136/bmjdrc-2019-001150

Wroe et al. 2018	presenting with symptoms consistent with depression or anxiety, or both, as indicated by either PHQ-9 score of 10 or above, or GAD-7 score of 8 or above, and a clinical assessment that indicated a presentation of depression or anxiety.		Excluded if their goals for therapy were not related to an improvement in depression or anxiety.
Chew et al. 2018	Included if presence of diabetes distress according to DDS (score of 3 or more). Excluded if any known psychiatric/psychological disorders that could impair judgement and memory.	Read or understand English or Malay; BP 140/90 mmHG or more; LDL level 2.6 or more.	
Chwastiak et al. 2018	Excluded if presence of cognitive impairment, current suicidality, homicidally.	BP>140/90; read English.	Excluded if grave disability that requires hospitalisation, cardiovascular event in last month, life expectancy less than a year.

Table S7- Frequency of studies with suboptimal glycaemic control as an inclusion criteria and HbA1c as a primary outcome

	Primary outcome= HbA1c		
**Inclusion criteria=Suboptimal HbA1c**	**Yes**	**No**	**Total**
**Yes**	8	3	11
**No**	25	34	59
**Total**	33	37	70

Table S8- Number of studies and arms included in the network meta-analyses for adults with type 2 diabetes.

Arm	N	%		Sample size
**CBT**	24	16·44	T	1268
**Counselling**	46	31·51	T	6105
**Usual care**	46	31·51	C	5954
**Attention control**	18	12·33	C	1297
**Self-help materials**	4	2·74	C	792
**IPT**	1	0·68	T	11
**Diabetes education**	1	0·68	C	46
**Waiting list control**	6	4·11	C	229
**Total**	146	100		15702

T=arm was defined as treatment arm and C= arm was defined as control group in original study. IPT=interpersonal therapy.

Table S9- Direct and indirect treatment effects (where indirect treatment effects were available) and the difference between them for adults with type 2 diabetes, including significance test for difference.

**Comparison**		**Direct**		**Indirect**		**Difference**		
		SMD	SE	SMD	SE	SMD	SE	p
Usual care	Attention control	-0·02	0·281	-0·032	0·083	0·012	0·293	0·966
Usual care	Self-help materials	-0·091	0·176	-0·412	0·171	0·321	0·246	0·192
CBT	Usual care	0·231	0·081	0·088	0·136	0·144	0·158	0·364
CBT	Attention control	0·035	0·126	0·275	0·118	-0·241	0·173	0·164
CBT	Self-help materials	-0·117	0·288	-0·048	0·153	-0·068	0·326	0·834
CBT	Waiting list control	0·352	0·209	0·266	0·207	0·086	0·295	0·770
Counselling	Usual care	0·188	0·053	0·259	0·151	-0·071	0·16	0·659
Counselling	Attention control	0·231	0·079	-0·044	0·141	0·274	0·162	0·091
Counselling	Self-help materials	-0·192	0·151	0·174	0·201	-0·366	0·252	0·146
Counselling	Waiting list control	0·274	0·191	0·36	0·224	-0·086	0·295	0·770
Attention control	Self-help materials	0·102	0·285	-0·317	0·15	0·419	0·322	0·193

Table S10- Summary of treatment effects compared with usual care assuming common heterogeneity estimate for all treatment design comparisons for adults with type 2 diabetes.

**Treatment**	**b**	**95% C.I.**	**SE**	**z**	**p**
Usual care	0				
CBT	-0·194	-0·33 to -0·057	0·069	-2·79	0·005
Counselling	-0·196	-0·292 to -0·099	0·049	-3·97	<0·001
Attention control	-0·031	-0·185 to 0·124	0·079	-0·39	0·698
Self-help material	-0·257	-0·499 to -0·015	0·123	-2·08	0·037
Waiting list control	0·114	-0·174 to 0·402	0·147	0·78	0·437

Winkley K, *et al. BMJ Open Diab Res Care* 2020; 8:e001150. doi: 10.1136/bmjdrc-2019-001150

Table S11- Summary of pairwise comparisons of all treatment assuming common heterogeneity estimate for all treatment design comparisons for adults with T2DM. SMD=SMD: Standardised mean difference

Treatment comparison		SMD	(95% C.I>)	SE	z	p
Usual care	CBT	-0.264	(-0.41 to -0.117)	0.075	-3.520	**<0.001**
Usual care	Counselling	-0.222	(-0.313 to -0.13)	0.047	-4.740	**<0.001**
Usual care	Attention control	-0.038	(-0.192 to 0.117)	0.079	-0.480	0.635
Usual care	Self-help materials	-0.243	(-0.479 to -0.007)	0.120	-2.020	**0.044**
Usual care	IPT	0.059	(-0.222 to 0.341)	0.144	0.410	0.679
Usual care	Structured diabetes	-0.160	(-0.475 to 0.154)	0.160	-1.000	0.318
Counselling	CBT	0.042	(-0.123 to 0.208)	0.084	0.500	0.616
Attention control	CBT	0.226	(0.034 to 0.418)	0.098	2.310	**0.021**
Self-help materials	CBT	0.021	(-0.251 to 0.293)	0.139	0.150	0.880
IPT	CBT	0.323	(0.009 to 0.638)	0.160	2.010	**0.044**
Structured diabetes	CBT	0.104	(-0.239 to 0.447)	0.175	0.590	0.553
Attention control	Counselling	0.184	(0.044 to 0.324)	0.071	2.580	**0.010**
Self-help materials	Counselling	-0.021	(-0.252 to 0.21)	0.118	-0.180	0.857
IPT	Counselling	0.281	(-0.01 to 0.572)	0.149	1.890	0.059
Structured diabetes	Counselling	0.061	(-0.239 to 0.362)	0.153	0.400	0.689
Self-help material	Attention control	-0.205	(-0.459 to 0.049)	0.129	-1.590	0.113
IPT	Attention control	0.097	(-0.207 to 0.401)	0.155	0.620	0.532
Structured diabetes	Attention control	-0.123	(-0.454 to 0.209)	0.169	-0.730	0.468
IPT	Self-help material	0.302	(-0.061 to 0.665)	0.185	1.630	0.103
Structured diabetes	Self-help material	0.083	(-0.296 to 0.461)	0.193	0.430	0.669
Structured diabetes	IPT	-0.219	(-0.637 to 0.199)	0.213	-1.030	0.305

Table S12- Probability to be the best treatment, mean rank and surface under the cumulative curve (SUCRA) for adults with type 2 diabetes derived from ranking probabilities.

Rank	Usual care	CBT	Counselling	Attention control	Self-help materials	Waiting list control
Best	0	22.4	18.8	0.1	58.1	0.6
MEAN RANK	4.9	2.2	2.2	4.5	1.8	5.5
SUCRA	0.2	0.8	0.8	0.3	0.8	0.1

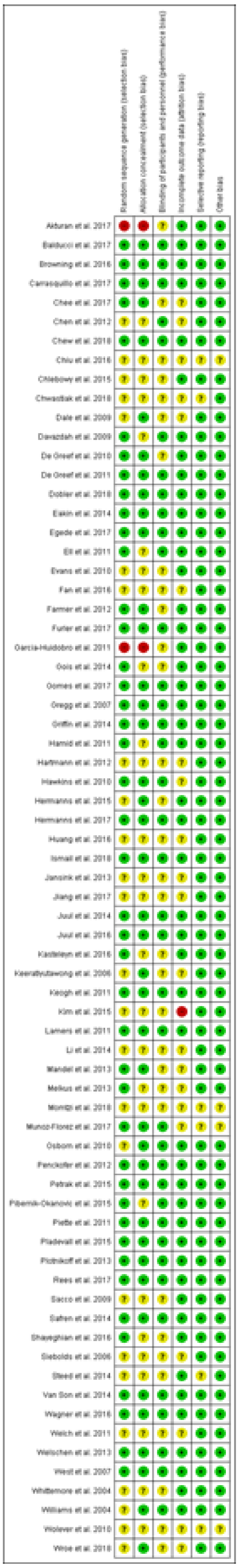

Figure S1- Risk of bias within RCTs of psychological interventions for adults with Type 2 diabetes.

Figure S2- Risk of bias domain assessment across psychological intervention RCTs for adults with Type 2 diabetes.

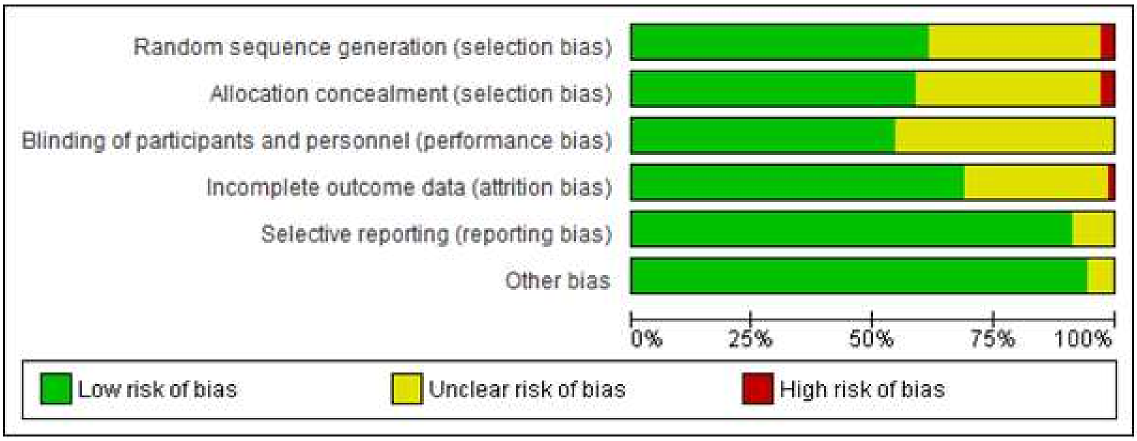

Figure S3- Network plots for reduce number of studies (N=143). Network plots of direct comparisons for the network meta-analysis for adults with type 2 diabetes.

Winkley K, *et al. BMJ Open Diab Res Care* 2020; 8:e001150. doi: 10.1136/bmjdrc-2019-001150

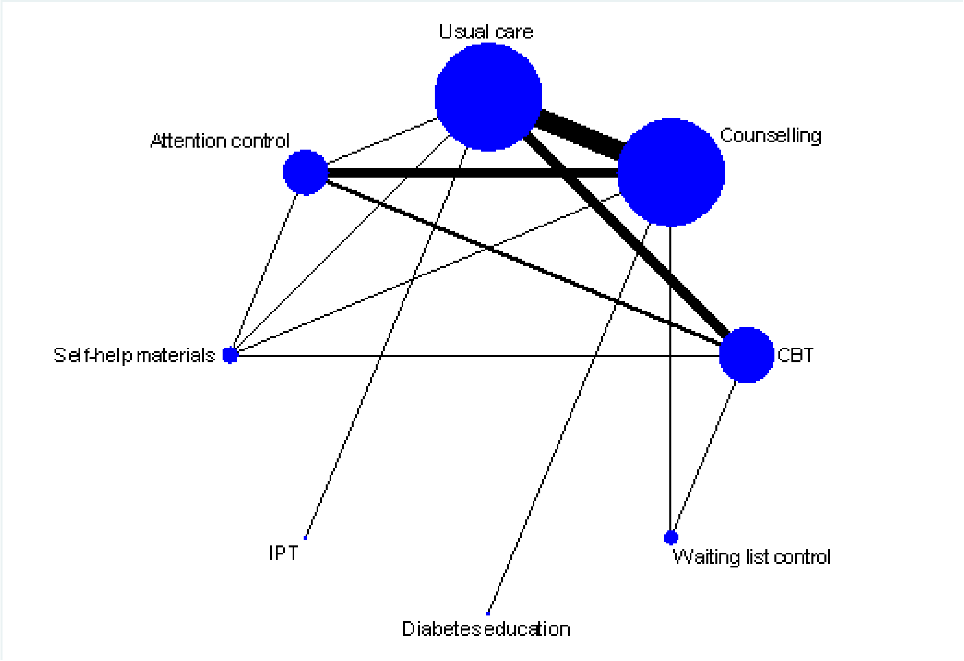

The width of the lines is proportional to the number of trials comparing each pair of treatments and the size of each node is proportional to the number of studies testing the specific treatment. It shows roughly how much information is available for each treatment and for each treatment comparison. IPT=interpersonal therapy, CBT=cognitive behavioural therapy.

Winkley K, *et al. BMJ Open Diab Res Care* 2020; 8:e001150. doi: 10.1136/bmjdrc-2019-001150
